# Supplementary material for: Metabolic response of a chemolithoautotrophic archaeon to carbon limitation
Source: mSystems. 2025 Sep 30;10(10):e00732-25. doi: 10.1128/msystems.00732-25 (PMC12542715; doi:10.1128/msystems.00732-25)
Supplement: Supplemental Material — Supplemental text and figures. [file msystems.00732-25-s0002.pdf]

# Supplementary Material for:

## Metabolic Response of a Chemolithoautotrophic Archaeon to Carbon Limitation

Logan H. Hodgskiss[1*], Melina Kerou[1], Zhen-Hao Luo[1], Barbara Bayer[2,3], Andreas Maier[4], Wolfram Weckwerth[5,6], Thomas Naegele[7*], Christa Schleper[1]

**Author affiliations:**

[1]Department of Functional and Evolutionary Ecology, Archaea Biology and Ecogenomics Unit, University of Vienna, Djerassiplatz 1, 1030 Vienna, Austria

[2]Department of Functional and Evolutionary Ecology, Bio-Oceanography and Marine Biology Unit, University of Vienna, Djerassiplatz 1, 1030 Vienna, Austria

[3]Centre for Microbiology and Environmental Systems Science, Division of Microbial Ecology, University of Vienna, Djerassiplatz 1, 1030 Vienna, Austria

[4]Department of Geography and Regional Research, Working Group Geoecology, University of Vienna, Josef-Holaubek-Platz 2, Vienna, 1090, Austria

[5]Department of Functional and Evolutionary Ecology, Molecular Systems Biology Unit (MOSYS), University of Vienna, Djerassiplatz 1, 1030 Vienna, Austria

[6]Vienna Metabolomics Center (VIME), University of Vienna, Djerassiplatz 1, 1030 Vienna, Austria

[7]Plant Evolutionary Cell Biology, Faculty of Biology, Ludwig-Maximilians-Universität Munich, Großhaderner Str. 2-4, 82152 Planegg-Martinsried, Germany

*Corresponding authors: logan.hodgskiss@univie.ac.at, thomas.naegele@lmu.de

**Key words:** ammonia-oxidation, archaea, carbon limitation, nitrogen cycle, oxidative stress

**Contents:**

Supplementary Discussion

Supplementary Materials and Methods

Supplementary Figures S1-S16

Supplementary Data (Dataset_S1, included Excel sheet)

# Supplementary Discussion

*Growth Effects of Pyruvate vs. Catalase and Carbon Balances of Conditions*

Cultures were harvested in late exponential phase when possible. Condition B (0.1 mM carbon with catalase) was harvested earlier than other cultures before it reached stationary phase due to a lack of carbon (Figure S2). The most limited condition (E) with 0 mM of supplied inorganic carbon presented cultures with an extended lag phase and very slow growth as measured by nitrite production (Figure 1). In these cultures, the only available inorganic carbon came from added pyruvate that was decarboxylated in the presence of hydrogen peroxide (Fig. 1C). The use of pyruvate or catalase as a ROS scavenger also produced a notable difference in the other limited cultures. All cultures of condition F (0.1 mM carbon with pyruvate) and condition B (0.1 mM carbon with catalase) were grown with 0.1 mM inorganic carbon. However, the use of pyruvate in condition F to counter ROS provided an additional release of $CO_2$. This slight difference resulted in F cultures being able to oxidize more ammonia than condition B, which entered stationary phase at ~1400 μM $NO_2^-$ (Figure S2). Cultures of conditions A (2 mM carbon with catalase), C (0.25 mM carbon with catalase), D (0.75 mM carbon with catalase), and G (0.25 mM carbon with pyruvate) all exhibited unimpaired growth.

Differences in carbon consumption were observed even among cultures with similar growth curves (Figure S3). Conditions with an excessive amount of carbon (conditions A and D; 2 mM and 0.75 mM, respectively) consumed a higher amount of carbon/nitrite produced (mol/mol) than cultures that were closer to the theoretical carbon threshold (conditions C and G) or under the threshold (conditions B, F, and E) (Figure S3). The higher amount of consumed carbon could have been incorporated into the carbon storage molecule polyhydroxybutyrate (PHB) or alternatively formed precipitates that would not be captured in the inorganic carbon measurements. Protein and DNA levels normalized to nitrite produced in cultures of the extremely carbon-limited condition E indicate a lower number of cells and a putative decoupling of ammonia oxidation and cell growth in extremely limited cultures (Figure S3) However, while there is a trend, variability within the data (i.e., condition C for DNA) and the lack of a strong reaction in wet biomass levels (with the exception of condition D) make this difficult to interpret. The similarities in wet biomass are likely due to the inability to measure such small amounts of cells with water present. A more thorough analysis using cell counts would be needed to confirm this observation.

*Proteins highly abundant in all conditions*

Enrichment analysis according to archaeal clusters of orthologous groups (arCOGS) (1) of these highly abundant shared proteins showed a high number of proteins involved in: energy production and conversion (arCOG C) and translation, ribosomal structure and biogenesis (arCOG J) (Figure S15). Proteins implicated in energy production included AmoB (ammonia monooxygenase enzyme subunit B), NirK, and subunits of ATP synthase (AtpA, AtpC, and AtpE). Numerous proteins involved in carbon metabolism were detected including aspartate semialdehyde dehydrogenase (Asd; carbon fixation cycle), glutamate dehydrogenase (GdhA; ammonium assimilation), malate dehydrogenase (Mdh; TCA cycle) and bifunctional fructose-1,6-bisphosphate/aldolase (Fbp; gluconeogenesis). Although these proteins represent the most highly abundant proteins in all conditions, the majority of them (89%) were also seen to change in relative abundance across conditions suggesting that they are still under regulatory control with respect to carbon concentration.

*Proteins with no statistical change across conditions*

Approximately 80.7% of the total detected proteins showed a change among conditions with 20.3% remaining constant. Proteins that did not change in response to carbon concentrations included ribosomal proteins and selected proteins involved in purine and pyrimidine metabolism but were not enriched for any arCOG category (Figure S15). AmoB, AmoY (a newly identified subunit of the archaeal ammonia monooxygenase (2)), and NirK were also included in this category underscoring their importance to cellular function regardless of carbon limitation. Other proteins involved in purine metabolism (PurU, PurA, AdkA, PurD, GuaA), pyrimidine metabolism (ThyX, PyrE, PyrF. PyrB), the non-oxidative pentose phosphate pathway (RpiA, Tal), and the conserved connection point between C-3 and C-4 metabolism (3) in AOA (PckA, ATP-dependent phosphoenolpyruvate carboxykinase) were also found to remain constant across the different growth conditions. Many of these are involved in carbon metabolism and the production of vital metabolites, i.e. nucleotides.

*Responses of P-II regulatory proteins to carbon limitation underscore roles in central metabolic regulation*

One possible regulatory mechanism that connects different metabolic pathways in the cell could come from the action of nitrogen regulatory P-II proteins. *Nitrososphaera viennensis* encodes for six P-II proteins in its genome (3), which are predicted to tightly control the flow of carbon and nitrogen in the cell. Five out of six were detected in the proteomes. Two of these

proteins (CnrC and CnrD), both of which increased under carbon limitation, are among the proteins with the highest relative abundance across all conditions (Figure S7). Their high abundance, coupled with an increase under carbon limitation, suggests that their regulatory functions help to increase the flow of carbon into the cell. Conversely, CnrB was decreased under carbon limitation, and GlnB almost completely disappeared from the detected proteome (Figure S7). A decrease in GlnB is unsurprising as its activity is known to inhibit the activity of acetyl-CoA carboxylase (Acc) in *Escherichia coli* (4). However, this also represents a unique response in *N. viennensis*, and perhaps other archaeal autotrophs when presented with carbon limitation. In bacteria and eukaryotes, a decrease in carbon metabolites initiates an increase in GlnB to prevent the storage of carbon in fatty acids by the activity of Acc (4–6). The opposite effect is observed here, as Acc is the key enzyme for fixing carbon in AOA rather than for storing excess carbon in fatty acids (lipids not commonly found in archaeal biomass). As such, the regulation of GlnB must be under a different mechanism than that found in other domains of life that rely on Acc for fatty acid synthesis. It is plausible that AOA rely on GlnB as a way to regulate Acc under carbon excess, but a unique regulation mechanism would still be needed to reverse this under carbon limitation. Alternatively, the consistent presence of GlnB within AOA genomes could point towards other functional roles outside of Acc inhibition. The need for AOA to decrease GlnB under carbon limitation would likely impact these other roles. The increased number of P-II proteins found within AOA, with presumably different ligand binding sites, may be a way to compensate for adverse effects of GlnB down-regulation.

*Involvement of NVIE_010650 in translation*

A structure-based search revealed that NVIE_010650 contains a PRC-barrel domain. These domains can have implications in both archaeal FtsZ cell division (7) and ribosome maturation (8). While *N. viennensis* encodes an FtsZ homolog, this was not detected in any of our datasets. The presence of Cdv proteins in the proteome (Dataset S1) correspond with previous data from *Nitrosopumilus maritimus* that AOA do not divide with an FtsZ-like system (9), thus ruling out a role of NVIE_010650 in cell division. Although significant differences can be seen in the amino acid alignments (Figure S16), the structural search found the closest protein candidates to likely be involved in ribosome maturation (Dataset S1).

*Core metabolism response to pyruvate addition*

A response at specific points within the core metabolism was also observed that could be explained by the presence of additional redox stress from reactive oxygen species (ROS) or

reactive nitrogen species (RNS) (Figures 4 and 7). Within gluconeogenesis, a redundancy in the conversion of glycerate-2P to glyerate-3P exists in *N. viennensis* with the proteins ApgM and GpmB both facilitating this step. While ApgM is higher in all conditions and responds to carbon limitation, the additional up-regulation of GpmB specifically under pyruvate would also direct more carbon to glycerate-3P, the precursor for serine and therefore cysteine, an amino acid that is commonly impacted by ROS/RNS stress and can have important implications when dealing with intracellular ROS (10). In support of this, a redundant putative cysteine synthase (CysM1) was also found within this cluster, suggesting a response of the cell to replace commonly damaged amino acids (i.e., cysteine). A slight up-regulation of aconitase (Aco) within the TCA cycle is also seen in pyruvate conditions which may also support the flow of carbon towards alpha-ketoglutarate and the assimilation of ammonium, a necessary step for amino acid production to replace damaged proteins and amino acids, including cysteine. The response of aconitase is supportive of the acquisition of a mitochondrial-type aconitase (Aco) in AOA (11) that is likely responsive to the redox status of the cell (11, 12) (Figures 4 and 7).

It is tempting to speculate that the pyruvate could be driving the slight up-regulation of Aco for carbon assimilation. An early study of *N. viennensis* showed incorporation of $C^{13}$ labeled pyruvate into biomass (5.85-9.54% of total carbon) along with a production of $C^{13}$ labeled $CO_2$ accounting for 6.46-7.09% of $CO_2$ in the culture headspace (13). It was later proven that hydrogen peroxide is detoxified by pyruvate leading to a release of $CO_2$ (14). The ROS detoxifying nature of pyruvate and the lack of an endogenous catalase in many AOA, including *N. viennensis*, explains the substantial growth rate increase that is stimulated by some organic acids (13). This information casts doubt on a mixotrophic nature of *N. viennensis*. The previously labeled biomass was likely the result of the cell fixing $C^{13}$ labeled $CO_2$ that had been cleaved from the fully $C^{13}$ labeled pyruvate as evidenced by its presence in the culture headspace. The direct incorporation of organic carbon into AOA biomass has been suggested for other clades based on genomic comparisons but has yet to be substantially confirmed (15). For these reasons, a direct incorporation of pyruvate into biomass is yet to be shown for *N. viennensis* and is not assumed to be happening in this study.

# Supplementary Methods

*Thermodynamic Model*

A thermodynamic model for the metabolism of *N. viennensis* was constructed based off of the Thermodynamic Electron Equivalents Model 2 (TEEM2) method (16).

The electron donor reaction was calculated as the oxidation of ammonia to nitrite. Ammonia was chosen for the half reaction instead of ammonium as it is assumed that ammonia, and not ammonium, is the true substrate for the AMO complex (17) that begins the ammonia oxidation pathway:

$$2H_2O + NH_3 \rightarrow NO_2^- + 7H^+ + 6e^- \qquad \textbf{Eq. 1}$$

The reaction was then normalized to one electron and arranged in the reducing direction:

$R_d$: $$\frac{1}{6}NO_2^- + \frac{7}{6}H^+ + e^- \rightarrow \frac{1}{3}H_2O + \frac{1}{6}NH_3 \qquad \textbf{Eq. 2}$$

Oxygen reduction was used as the half reaction for the electron acceptor:

$$4e^- + 4H^+ + O_2 \rightarrow 2H_2O \qquad \textbf{Eq. 3}$$

This equation was also normalized to one electron:

$R_a$: $$e^- + H^+ + \frac{1}{4}O_2 \rightarrow \frac{1}{2}H_2O \qquad \textbf{Eq. 4}$$

Carbon and nitrogen measurements of washed *N. viennensis* determined the C:N molar ratio to be 3.85:1. Although molar ratios of oxygen and hydrogen were not determined, a biomass composition of $C_{3.85}H_{6.69}O_{1.78}N$ was used. This was based off of values for *Escherichia coli* that had the same C:N molar ratio of 3.85:1 (18) (pg. 129). The biomass composition was used with the following formula to estimate a half reaction for biomass formation:

$R_c$: $$\frac{n}{d}CO_2 + \frac{c}{d}NH_3 + H^+ + e^- \rightarrow \frac{1}{d}C_nH_aO_bN_c + \frac{2n-b}{d}H_2O \qquad \textbf{Eq. 5}$$

where $d = 4n + a - 2b - 3c$ .

This equation is modified from Rittmann and McCarty (18) (pg. 137). Typically, a generic cell synthesis equation would include $NH_4^+$ rather than $NH_3$ to represent a nitrogen source. As the biomass nitrogen source ($NH_4^+$) and electron donor ($NH_3$) in AOA come from the same $NH_4^+/NH_3$ pool, the equation was modified to use $NH_3$ as the representative species of the nitrogen pool based on the physiological observation that the vast majority of nitrogen

consumed by *N. viennensis* is used for nitrite production and would therefore be utilized in the form of $NH_3$. The original equation also includes $HCO_3^-$ to balance the positive charge of ammonium. Therefore, $CO_2$ was used in place of $HCO_3^-$ as a counter-charge balance was not needed. The use of $NH_3$ (and therefore $CO_2$) in $R_c$ (Eq. 5) is also consistent with the use of $NH_3$ in $R_d$ (Eq. 2) and accurately reflects the splitting of the same nitrogen pool into biomass and nitrite when the equations are combined (Eq. 9, see below). Although the actual substrates for AOA biomass are $NH_4^+$ and $HCO_3^-$, the use of $NH_3$ and $CO_2$ are more consistent when balancing the overall chemical reaction and do not alter the biomass equation as each contains the same amount of nitrogen and carbon respectively. Utilizing the estimated biomass composition and the modified equation gives the following:

$R_c$: $$\frac{3.85}{15.53}CO_2 + \frac{1}{15.53}NH_3 + H^+ + e^- \rightarrow \frac{1}{15.53}C_{3.85}H_{6.69}O_{1.78}N + \frac{5.92}{15.53}H_2O \quad \textbf{Eq. 6}$$

Once the necessary reactions were obtained, $\Delta G^{0'}$ values were calculated. Gibbs free energy was calculated for a temperature of 25°C and pH of 7.0. These are not the exact temperature of the growth conditions for *N. viennensis* (42°C and pH ~7.2 respectively). However, standard conditions were used as these are the parameters used to calculate the Gibbs free energy for the reduction of oxygen.

Oxygen reduction: $\Delta G_a^{0'} = -78.72\frac{kJ}{mol\ e^-}$ [29]

As this is an empirical value based on a hydrogen potential with platinum, it is not readily adjustable. Values were also found for differing temperatures using the WORM portal and later tested to verify results (see below). For consistency, the same parameters (25°C and pH of 7.0) were used for the calculation of Gibbs free energy for the half reaction of ammonia to nitrite. Gibbs free energy of formation was used for the participating chemical species:

$H_2O_{(l)}$: $\Delta_f G_{H_2O} = -237.13\ kJ/mol$ (19)

$NH_{3(aq)}$: $\Delta_f G_{NH_3} = -26.50\ kJ/mol$ (19)

$NO_2^-{}_{(aq)}$ : $\Delta_f G_{NO_2^-} = -8.9\frac{kcal}{mol} = -37.24\ kJ/mol$ (Using 4.184 kJ/kcal.) (20)

$$\Delta_r G^0 = [products] - [reactants] \quad \textbf{Eq. 7}$$

Using Equation 2:

$$\Delta_r G^0 = \frac{1}{3}\left(-237.13\frac{kJ}{mol}\right) + \frac{1}{6}\left(-26.50\frac{kJ}{mol}\right) - \frac{1}{6}\left(-37.24\frac{kJ}{mol}\right) = -77.25\frac{kJ}{mol}$$

To adjust for pH of 7.0:

$$\Delta G^{0\prime} = \Delta G^0 + RT\ln\left(\frac{[H_2O]^{\frac{1}{3}}[NH_3]^{\frac{1}{6}}}{[NO_2^-]^{\frac{1}{6}}[H^+]^{\frac{7}{6}}}\right),\ [H^+]=10^{-7} \qquad \textbf{Eq. 8}$$

$$\Delta G^{0\prime} = -77.25\,\frac{kJ}{mol} + \left(0.008314\,\frac{kJ}{K\cdot mol}\right)(298\,K)\ln\left(\frac{[1]^{\frac{1}{3}}[1]^{\frac{1}{6}}}{[1]^{\frac{1}{6}}[10^{-7}]^{\frac{7}{6}}}\right) = -30.66\,\frac{kJ}{mol}$$

Ammonia oxidation in reducing direction: $\Delta G_d^{0\prime} = -30.66\,\frac{kJ}{mol\ e^-}$

For calculation of model:

$$R = f_e^0 R_a + f_s^0 R_c - R_d \qquad \textbf{Eq. 9}$$

$$f_s^0 + f_e^0 = 1 \ \textbf{Eq. 10}$$

$$f_s^0 = \frac{1}{1+A} \qquad \textbf{Eq. 11}$$

$$A = -\frac{\Delta G_s}{\varepsilon \Delta G_r} \qquad \textbf{Eq. 13}$$

$$\Delta G_r = \Delta G_a^{0\prime} - \Delta G_d^{0\prime} - \frac{q}{p}\Delta G_{xy} \textbf{Eq. 14}$$

$$\Delta G_s = \frac{(\Delta G_{fa} - \Delta G_d^{0\prime})}{\varepsilon^m} + \frac{(\Delta G_{in} - \Delta G_{fa})}{\varepsilon^n} + \frac{\Delta G_{pc}}{\varepsilon} \qquad \textbf{Eq. 15}$$

where $\Delta G_a^{0'}$ is the Gibbs free energy for the electron acceptor half reaction, $\Delta G_d^{0'}$ is the Gibbs free energy for the electron donor half reaction, q represents the number of monooxygenase reactions per substrate, p is the number of electron equivalents per mole of substrate, $\Delta G_{xy}$ is the reduction potential for NADH oxidation (representative of the energy input for the monooxygenase reaction; -219.2 kJ/mol (16)), $\Delta G_{fa}$ is the reduction potential for a formaldehyde half reaction, $\Delta G_{in}$ is the Gibbs free energy for the reduction potential of acetyl-CoA (representative of carbon activation for autotrophs; 30.9 kJ/mol (16)) $\Delta G_{pc}$ is the Gibbs free energy for intermediate conversion to cells, ε is the energy transfer efficiency, m is 1 if $\Delta G_{fa}$ is greater than 0 and equal to n otherwise, and n is 1 if ( $\Delta G_{in}$ - $\Delta G_d^{0'}$)>0 and m=n (16). The value $f_e^0$ represents the fraction of electron equivalents used for energy and the value $f_s^0$ represents the fraction of electron equivalents used for biomass synthesis.

In the case of *N. viennensis*, $\Delta G_{fa}$ =0 as no formaldehyde is involved and ( $\Delta G_{in}$ - $\Delta G_d^{0'}$)>0. This causes m=n=1. The Equation 13 for $\Delta G_s$ therefore simplifies to:

$$\Delta G_s = \frac{\Delta G_{in} - \Delta G_d^{0\prime} + \Delta G_{pc}}{\varepsilon} \quad \textbf{Eq. 16}$$

The value of p is equal to 6 (6 electrons per ammonia, Eq. 1), q is 1 (one monooxygenase reaction per ammonia), and $\Delta G_{pc}$ is the energy to synthesize cells:

$$\Delta G_{pc} = \frac{\left(molecular\ weight\ of\ biomass\ \frac{g}{mol}\right)\left(energy\ per\ gram\ of\ biomass\ \frac{kJ}{g}\right)}{\left(electron\ equivalents\ per\ biomass\ \frac{eeq}{mol}\right)} \quad \textbf{Eq. 17}$$

Molecular weight of $C_{3.85}H_{6.69}O_{1.78}N$ = 95.37 g/mol

Energy per gram of biomass=3.33 kJ/g (18 (pg. 160), 21)

Electron equivalents per biomass= 15.53 eeq/mol (Equation 6)

Input values for Equation 17:

$$\Delta G_{pc} = \frac{\left(95.37\frac{g}{mol}\right)\left(3.33\ \frac{kJ}{g}\right)}{\left(15.53\ \frac{eeq}{mol}\right)} = 20.45\ \frac{kJ}{eeq}$$

The energy transfer efficiency, ε, is estimated to be 0.57 as observed for other autotrophs (16).

Input values for Equation 16:

$$\Delta G_s = \frac{30.9\frac{kJ}{mol} - \left(-78.82\frac{kJ}{mol}\right) + 20.4\frac{kJ}{mol}}{0.57} = 228.37\frac{kJ}{mol}$$

Input values for Equation 14:

$$\Delta G_r = -79.82\frac{kJ}{mol} - \left(-30.66\frac{kJ}{mol}\right) - \frac{1}{6}\left(-219.2\frac{kJ}{mol}\right) = -11.63\frac{kJ}{mol}$$

Input values for Equation 13:

$$A = -\frac{228.37\frac{kJ}{mol}}{0.57\left(-11.63\frac{kJ}{mol}\right)} = 34.44\frac{kJ}{mol}$$

Input values for Equation 11:

Fraction of electron equivalents used for biomass synthesis:

$$f_s^0 = \frac{1}{1 + 34.44} = 0.028$$

Input values for Equation 10:

Fraction of electron equivalents used for energy:

$$f_e^0 = 1 - f_s^0 = 1 - 0.028 = 0.972$$

Input values and equations ($R_a$ as Eq. 4; $R_d$ as Eq. 2; $R_c$ as Eq. 6) for overall reaction (Equation 9):

$$R = f_e^0 R_a + f_s^0 R_c - R_d$$

$$f_e^0 R_a:\ 0.972\left(e^- + H^+ + \frac{1}{4}O_2 \rightarrow \frac{1}{2}H_2O\right); 0.972e^- + 0.972H^+ + 0.243O_2 \rightarrow 0.486H_2O$$

$$f_s^0 R_c: 0.028\left(\frac{3.85}{15.53}CO_2 + \frac{1}{15.53}NH_3 + H^+ + e^- \rightarrow \frac{1}{15.53}C_{3.85}H_{6.69}O_{1.78}N + \frac{5.92}{15.53}H_2O\right);$$

$$0.007CO_2 + 0.002NH_3 + 0.028H^+ + 0.028e^- \rightarrow 0.002C_{3.85}H_{6.69}O_{1.78}N + 0.011H_2O$$

$$-R_d; -\left(\frac{1}{6}NO_2^- + \frac{7}{6}H^+ + e^- \rightarrow \frac{1}{3}H_2O + \frac{1}{6}NH_3\right);$$

$$0.333H_2O + 0.167NH_3 \rightarrow 0.167NO_2^- + 1.167H^+ + e^-$$

Combine all equations and normalize to 1 mol of ammonia:

$$1.411O_2 + 0.064CO_2 + NH_3 \rightarrow 0.953H_2O + 0.017C_{3.85}H_{6.69}O_{1.78}N + 0.983NO_2^- + 0.983H^+$$

**Eq. 18**

Equation 18 normalized to 2 mol ammonia:

$$2.821O_2 + 0.128CO_2 + 2NH_3 \rightarrow 1.906H_2O + 0.033C_{3.85}H_{6.69}O_{1.78}N + 1.967NO_2^- + 1.967H^+$$

**Eq. 19**

The experimental design was guided by Eq. 19 calculated at a temperature of 25°C and pH of 7.0. Thermodynamic parameters for temperatures and pH more reflective of the experimental growth conditions were calculated by finding the Gibbs free energy (ΔG°) at different temperatures for the ammonia half reaction (Eq. 2) and oxygen reduction (Eq. 4) and from the Water-Organic-Rock-Microbe (WORM) portal (22, 23). These values were then corrected for pH using Eq. 20 and Eq. 21 respectively to find the final ΔG°' used in Eq. 14 and Eq. 15 (Supp. Dataset S1, Table_S19).

$$\Delta G^{0\prime} = \Delta G^0 + RT\ln\left(\frac{[H_2O]^{\frac{1}{3}}[NH_3]^{\frac{1}{6}}}{[NO_2^-]^{\frac{1}{6}}[H^+]^{\frac{7}{6}}}\right) \quad \text{Eq. 20}$$

$$\Delta G^{0\prime} = \Delta G^0 + RT\ln\left(\frac{[H_2O]^{\frac{1}{2}}}{[O_2\ ]^{\frac{1}{4}}[H^+]}\right), \quad \text{Eq. 21}$$

At a temperature of 42°C and a pH of 7.2, the predicted C/N ratio was found to be 0.086 mol/mol. With a supplied ammonia concentration of 2 mM, this would resulted in a new threshold of 0.171 mM of inorganic carbon. While higher than the original model prediction of 0.128 mM of inorganic carbon, this threshold still supports the experimental design.

*Experimental Setup for Carbon Conditions*

With a nitrogen input concentration of 2 mM (ammonia/ammonium), a theoretical carbon threshold of 0.128 mM was used for the experimental design of growth curves with varying levels of inorganic carbon: 2 mM carbon with catalase (A); 0.75 mM carbon with catalase (D) 0.25 mM carbon with pyruvate (G); 0.25 mM carbon with catalase (C); 0.1 mM carbon with pyruvate (F); 0.1 mM carbon with catalase (B); and 0 mM carbon with pyruvate (E). This spread of carbon concentrations produced a gradient from high to low amounts of available inorganic carbon (Figure 1A). The reaction of pyruvate with ROS would create

additional available carbon for the cell. In the case of the 0 mM inorganic carbon concentration, this created a slow release of carbon substrate over time. The pyruvate conditions with 0.25 mM and 0.1 mM of added carbon would therefore have slightly more available carbon than their catalase counterparts. The combination of all conditions gives a gradient of carbon concentrations starting from 2 mM and dropping down to just above 0 mM.

*Carbon and Nitrogen Content of Cell*

Cells were grown under standard conditions with pyruvate and harvested in late exponential growth phase by centrifugation (16,000 x*g* at 4°C for 1 h). Cell pellets were washed twice with FWM (no additions of bicarbonate or ammonium), dried for 1h (vacuum concentrator) and stored in a desiccator until further processing. Subsequently, cell pellets were weighed and packed into tin capsules prior to being analyzed on a CHNS elemental analyzer (Vario MICRO cube, Elementar). The instrument was calibrated with acetanilide following manufacturer protocols.

*Culture Conditions*

Cultures of *N. viennensis* were grown in closed 1 liter pressure safe Schott bottles closed with a grey bromobutyl rubber stopper (GL45 Duran) with 580 mL of fresh water medium (1g/L NaCl, 0.4 g/L $MgCl_2 \cdot 6H_2O$, 0.1 g/L $CaCl_2 \cdot 2H_2O$, 0.2 g/L $KH_2PO_4$, 0.5 g/L KCl). The choice of rubber stopper is critical as *N. viennensis* will not grow when certain other rubber stoppers are used. The following was added to FWM: 600 µL trace element solution (100 mM HCl (~12.5M), 0.5 mM $H_3BO_3$, 0.5 mM $MnCl_2 \cdot 4H_2O$, 0.8 mM $CoCl_2 \cdot H_2O$, 0.1 mM $NiCl_2 \cdot 6H_2O$, 0.01 mM $CuCl_2 \cdot H_2O$, 0.5 mM $ZnSO_4 \cdot 7H_2O$, 0.15 mM $Na_2MoO_4 \cdot 2H_2O$; autoclaved, stored in dark), 600 µL vitamin solution (0.02 g/L biotin, 0.02 g/L folic acid, 0.1 g/L pyridoxine HCl, 0.05 g/L thiamine HCl, 0.05 g/L riboflavin, 0.05 g/L nicotinic acid, 0.05 g/L DL pantothenic acid, 0.05 g/L P aminobenzoic acid, 2 g/L choline chloride, 0.01 g/L vitamin B12; adjusted pH to 7 with KOH, filter sterilized, stored in dark), 600 µL 7.5 mM FeNa-EDTA (pH 7), and 6 mL HEPES buffer solution (24 g/L NaOH, 119.2 g/L HEPES; pH ~7.6, filter sterilized).

For all cultures, kanamycin was added to a final concentration of 100 mg/mL and $NH_4Cl$ was added to a final concentration of 2 mM.

A catalase stock solution was made by dissolving catalase from bovine liver (Sigma Aldrich, powder, 2000-5000 units/mg protein (average of 3500 unit/mg)) in a potassium

phosphate buffer solution (0.6528 g/L $KH_2PO_4$, 7.83 g/L $K_2HPO_4$, pH adjusted to 7.0) at 1 mg/mL. Catalase was filter sterilized before use and added at 50 U/mL final concentration as previously described (14). Cultures grown with catalase were observed to have some translucent flocs due to the addition of catalase. These flocs formed upon addition of catalase and were therefore not attributed to be a product of cell growth. Cultures using pyruvate as a ROS scavenger had pyruvate added to a final concentration of 0.3 mM. The concentration of 0.3 mM pyruvate was chosen as this would provide more than double of the necessary inorganic carbon if all pyruvate was decarboxylated.

Inorganic carbon was added from freshly made solutions of filter sterilized 0.5M sodium bicarbonate. Based on preliminary tests amounts of added bicarbonate are overestimated (i.e. a culture with assumed 2 mM of bicarbonate is actually 1.67 mM bicarbonate). This discrepancy is likely due to the exchange of carbon dioxide between the gas and aqueous phases. The actual concentration of a freshly prepared 0.5 M stock solution was determined to be 0.415 mM. This concentration was used to calculate the amount of stock solution to add to cultures. Therefore, for 600 mL cultures, the following amounts of freshly made 0.5M sodium bicarbonate solution was added: 2 mM carbon culture was supplied with 2.87 mL, a 0.75 mM culture was supplied with 1.08 mL, a 0.25 mM culture was supplied with 0.36 mL, and a 0.1 mM culture was supplied with 0.14 mL.

All cultures were inoculated with 1.5 mL (0.25% inoculum) of an actively growing culture in late exponential phase. Final volume after additions and inoculation comes to approximately 600 mL.

After inoculation, the headspace of cultures was flushed with an artificial gas mixture of 80% nitrogen 20% oxygen to remove any residual carbon dioxide in the head space. Immediately after flushing the head space, a water sample was taken from the aqueous phase to determine the total starting carbon amount (see below). Cultures were then grown in a 42°C incubator on a shaker set at 80 rpm.

Cultures were tracked by taking periodic nitrite measurements. Cultures were harvested when late exponential phase was reached but before entering stationary phase. At the end of a growth curve, samples were taken for gas analysis and inorganic carbon analysis of liquid. One milliliter was collected and spun down for 1 hour at 16,000xg and 4°C. The supernatant was removed and the pellet was stored at -70°C until DNA could be extracted. The rest of the culture was spun down in 250-300 mL volumes using a large Sorvall centrifuge for

45 minutes at 16,100xg at 4°C. Supernatant was poured off and the pellet was resuspended in remaining media and transferred to a new 1.5 mL Eppendorf tube. Cells were then concentrated by centrifuging at 16,100xg for 30 minutes and 4°C. As much supernatant as possible was removed without disturbing the cell pellet. Eppendorf tubes were weighed before and after cells were added to obtain an approximated wet biomass weight. Pellets were then frozen at -70°C until further metabolite and protein extraction.

*Ammonium Measurements*

Ammonium was measured colorimetrically. 200 µL of sample was mixed with 400 µL of fresh water medium (FWM) in an Eppendorf tube. 300 µL of color reagent (5.18 mM sodium salicylate, $2.15 \times 10^{-5}$ M sodium nitroprusside, 0.1 M NaOH; freshly prepared) was added followed by 120 µL of oxidation solution ($3.01 \times 10^{-5}$ M dichloroisocyanuric acid; freshly prepared). Samples were shaken and incubated in the dark for 30 minutes. After 30 minutes, 200 µL of sample were pipetted into wells in a micro titer plate and absorbance was measured at 660 nm using a Tecan-Sunrise plate reader. Standard curves were made using a stock solution of 1 mM $NH_4Cl$.

*Nitrite Measurements*

Nitrite was measured colorimetrically. 10 or 20 µL of sample was mixed with 790 or 780 µL fresh water medium (FWM) respectively and 200 µL of a sulfanilamide/NED reagent (150 mL ortho-phosphoric acid, 10 g sulfanilamide, 0.05 g α-naphtylethylendiamindihydrochloride in 1 L water; stored at 4°C in the dark) in a 1 mL Eppendorf tube. Samples were shaken and then stored in the dark for at least 10 minutes. After 10 minutes, 200 µL of each sample was pipetted into wells of a micro titer plate and absorbance was measured at 545 mM using a Tecan-Sunrise plate reader. Standard curves were made from a stock solution of 1 mM $NaNO_2$ (i.e. 1 mM $NO_2^-$ : 20 µL stock + 780 µL FWM; 0.8 mM $NO_2^-$ : 16 µL stock + 784 µL; 0.6 mM $NO_2^-$ : 12 µL stock + 788 µL; etc.).

*Dissolved Inorganic Carbon Analysis*

10 mL of sample were taken at the beginning (sterile, with flame and needle) and end of each culture to determine starting and ending dissolved inorganic carbon (DIC) concentration in the aqueous phase. Samples were filtered to remove cells and stored at -70°C until the inorganic carbon could be measured. DIC data were measured using a Shimadzu TOC -LCPH analyzer equipped with a DIC reaction vessel containing a reaction solution of

phosphoric acid of about 25% (weight %) and an ASI-L autosampler. The samples were injected into the reaction vessel where all inorganic carbon is converted to carbon dioxide which in the following is volatized by the sparging process (synthetic air, carbon dioxide free gas) and detected by a NDIR detector. Each sample is measured three times with an injection volume of 100 micro-liter each. The final results are the corresponding mean values of the three injections per sample.

*Gas Chromatography Analysis*

At the time of harvest, head space pressure was measured and 40-50 mL of head space gas was removed using a 50 mL syringe and transferred to a glass 120 mL serum bottle pre-filled with $CH_4$ at 1 atm. Mixed gas samples were then analyzed using an Agilent Gas Chromatograph (Agilent 7890A GC, Agilent Technologies, Santa Clara, CA, USA) using a thermal conductivity detector. Gases were separated at 170°C using helium as the carrier gas. The reference flow setting was 10 mL/min. The makeup flow was set to 1 mL/min.

The following gases were used: $H_2$, $CO_2$, $N_2$, $CH_4$, and $H_2/N_2/CO_2$ (mix ratio 7:1:1). All gases were from Air Liquide GmbH, Schwechat, Austria. The standard test gas used for gas chromatography (GC) comprised the following composition: 0.01% volume $CH_4$ and 0.08% volume $CO_2$ in $N_2$ (Messer GmbH, Wien, Austria).

*Carbon Balance*

The total amount of carbon consumed by each culture can be approximated by the equation:

$$C_{con} = C_{avail} - C_{aq} - C_g \qquad \textbf{Eq. 20}$$

where $C_{con}$ is total carbon consumed, $C_{avail}$ is amount of available inorganic carbon, $C_{aq}$ is the aqueous inorganic carbon concentration at the end of the experiment, and $C_g$ is the gaseous inorganic carbon concentration at the end of the experiment. $C_{avail}$ can be calculated by:

$$C_{avail} = C_{aq,0} + C_{g,0} + C_{pyr} \quad \textbf{Eq. 21}$$

where $C_{aq,0}$ is the initial aqueous inorganic carbon concentration, $C_{g,0}$ is the initial gaseous inorganic carbon concentration, and $C_{pyr}$ is the amount of inorganic carbon contributed from the interaction of pyruvate and ROS. In all cultures, the $C_{g,0}$ is 0 as the head space of the

cultures is flushed while $C_{aq,0}$ is represented by the initial IC measurement taken. In catalase cultures, $C_{pyr}$ is 0, while in pyruvate cultures, $C_{pyr}$ is estimated by:

$$C_{pyr} = [NO_2^-] * \frac{0.005\ mMH_2O_2}{0.5\ mM\ NH_4^+} * \frac{1\ mM\ NH_4^+}{0.983\ mMNO_2^-} * \frac{1\ mM\ CO_2}{1\ mM\ H_2O_2} \qquad \textbf{Eq. 22}$$

where $[NO_2^-]$ represents the mM of nitrite produced by the culture. The ratio of 0.0045 mM $H_2O_2$/ 0.5 mM $NH_4^+$, is based off estimates of hydrogen peroxide production of *N. viennensis* from supplementary material in Kim et al. (2016) (14), 1mM $NH_4^+$/0.983 mM $NO_2^-$ is based off of the growth equation calculated in this manuscript, and 1 mM $CO_2$/1 mM $H_2O_2$ is based off the assumption of stoichiometric release of carbon dioxide from pyruvate as it interacts with hydrogen peroxide.

*DNA Extraction*

DNA was extracted from 1 mL of each culture taken at the time of harvest using the NucleoSpin Soil kit from Machery-Nagel and by following instructions for genomic DNA extraction from soil using lysis buffer SL1. Extracted DNA was eluted into 60 µL of elution buffer SE (5 mM Tris/HCl, pH 8.5). Concentrations were measured using a Qubit DNA Assay with 5 µL (high sensitivity protocol).

*Combined Protein and Metabolite Extraction*

Methods of a combined protein and metabolite extraction were followed based on those of Ott et al. (2019) (24). Cell pellets were thawed on ice and resuspended in 500 µL methanol:chloroform:water (MCW, 2.5:1:0.5). Water for all extractions came from a MilliporeSigma Milli-Q Reference A+ System (MilliQ water). Cells and undissolved pellet were transferred to a Lysing Matrix B tube with Lysing Matrix B (MP Biomedical) filled to 2-3 mm above the cone shape at the bottom of the tube. As an internal control and extraction standard, pentaerythritol (PE) and phenyl-β-glucopyranoside (PGP) (5 µL of 1 mM solution for each) were spiked into each sample. Cells were lysed with FastPrep-24 homogenizer (MP Biomedical) for 30 seconds at a velocity of 4 m/s. After lysing, samples were cooled for 2 minutes on ice and then spun down for 2 minutes at 16,100 xg and 4°C. Supernatant was collected and saved after centrifugation. Cell lysis and centrifugation with the remaining sand pellet was repeated with 250 µL of MCW. After centrifugation, the supernatant was added to the previously collected supernatant for each sample. 250 µL of 80% ethanol was added to the remaining sand pellet. Samples were briefly vortexed and then incubated on a heat block at 80°C and 500 rpm for 30 minutes. After incubation, samples were spun down for 2 minutes at

16,100xg and 4°C. The ethanol supernatant was then collected and combined with the previously collected supernatant. Lysis tubes containing protein, cell debris, and the sand pellet were frozen at -70°C until protein extraction could be performed. 400 µL of water and 100 µL of chloroform were added to each of the collected supernatant tubes. Supernatant samples were then centrifuged for 5 minutes at 16,100xg and 4°C to separate phases. The upper phase (methanol, water, and polar metabolites) was collected into a new tube and dried using a step-wise pressure procedure. Dried samples were stored at -70°C until derivatization.

*Metabolite Derivatization and GC-MS Analysis*

Dried and frozen samples were allowed to acclimate to room temperature (~20 minutes). Under a fume hood, samples were dissolved in 20 µL of methoximation reagent (40 mg methoxyaminhydrochloride in 1 mL pyridine). Samples were then incubated for 90 minutes at 30°C and 900 rpm. Following incubation, 80 µL of silylation mix (1 mL N-methyl-N-trimethylsilyltrifluoroacetamid spiked with 30 µL of a mix of even-number alkanes (C10-C40)) was added to each sample and samples were incubated for 30 minutes at 37°C and 900 rpm. Following incubation, samples were centrifuged for 2 minutes at 14,000xg. Supernatant was then transferred to gas chromatography microvials and sealed with crimp caps.

Samples were analyzed in three separate runs in split-less injection mode on a Pegasus® BT GC-TOF-MS (LECO Corporation, St. Joseph, MI, USA) with a standard curve of selected metabolites for absolute quantification. Loaded volume for each sample was 1 µL. Metabolites were separated using a Rxi-5 ms column (30 m length, 0.25 mm diameter, 0.25 µm film; Restek, Centre County, PA, USA). The carrier gas as helium with a flow rate of 1 mL/min. Injection was done using a S/SSL injector with an injection temperature of 230°C. The column temperature started with 70°C for 1 minute and then followed a ramp to 340°C with at heating rate of 9°C/min. Temperature was held at 340°C for 15 minutes at the end. For the mass spectrometer, ion source and transfer line temperature was 250°C and acquisition delay was set to 300s. Masses were recorded in the range of 50-600 *m/z* with an acquisition rate of 10 spectra/sec.

Resulting peaks from selected metabolites were manually selected using LECO ChromaTOF software. Selected metabolites were absolutely quantified and normalized based on added extraction standards (see Data Analysis).

*Protein Extraction*

Sand pellets containing cellular debris and proteins were thawed on ice. Once thawed, sand pellets were washed with 500 µL of methanol. Samples were then centrifuged for 5 minutes at 16,100xg and 4°C. Methanol supernatant was then discarded and pellets were allowed to air dry under a fume hood.

To begin the extraction, 1 mL of TRIzol reagent (Thermo Fisher) was added to each pellet and mixed with the sand through gently pipetting. Samples were then incubated at room temperature for 15 minutes. Following incubation, 100 µL of chloroform was added and tubes were inverted 5 times. Samples then incubated at room temperature for three minutes. Following the incubation with chloroform, samples were centrifuged at 16,100 xg for 2 minutes at 4°C to separate phases. The lower phase, containing the proteins, was removed to a new lo-bind protein Eppendorf tube. 550 µL of water (or a 1:1 ratio with chloroform phase) was added to each sample. Tubes were inverted 5x and incubated at room temperature for 3 minutes. After incubation, tubes were centrifuged for 2 minutes at 16,100xg and 4°C. The apolar (bottom phase) was transferred to a new 2 mL low-bind protein Eppendorf tube. 1.5mL of ice cold 0.1 M $NH_4CL$ in methanol with 0.5% β-mercaptoethanol was added to each sample. Samples were then incubated on ice for 1-4 hours before being stored at -20°C overnight to facilitate protein precipitation.

*Protein Washing*

After overnight incubation at -20°C, samples were centrifuged for 15 minutes at 16,100xg and 4°C. The supernatant was discarded and 1.8 mL of ice cold methanol was added to each sample. Pellets were sonicated in an ice water bath with a Transsonic 700/H water bath (Elma) sonicator for 10-15 minutes or until pellets were completely dissolved. Samples were then centrifuged for 10 minutes at 16,100xg and 4°C. Supernatant was discarded and the washing and sonication steps were repeated with another 1.8 mL of ice cold methanol. After removal of the methanol supernatant from the second washing step, 1.8 mL of ice cold acetone was added to each sample. Pellets were again suspended in an ice water bath via sonication and then centrifuged for 15 minutes at 16,100xg and 4°C. The acetone supernatant was removed and pellets were air dried in a fume hood for 5-10 minutes (while avoiding over-drying). Once dry, proteins were stored at -70°C until further processing.

*Protein Digestion*

Dried protein pellets were resuspended in 500 µL of extraction buffer (8M urea in 50 mM HEPES, pH 7.8). Samples were then incubated for 30 minutes on a shaker at 4°C and 900 rpm. After incubation, protein concentrations were measured using the Bradford assay (Bio-Rad Cat. No. 500-0006) using a pre-made standard curve with bovine serum albumin (BSA) and an absorbance of 545 nm. The volume to get 25 µg of protein was calculated and taken for each sample and put into new low-bind protein Eppendorf tubes. Extraction buffer was added to each sample to reach a final volume of 220 µL. 5.64 µL of 200 mM dithiothreitol (DTT) was added to each sample and samples were then incubated for 45 minutes at 37°C and 700 rpm. Next, 2.26 of freshly prepared 1 M of iodoacetamide (IAA) was added and samples were incubated for 60 minutes at 30°C and 700 rpm. After the IAA incubation, a final amount of 6.27 µL of 200 mM DTT was added and samples were again incubated in the dark at room temperature for 15 minutes. Next 5 µL of 0.1 µg/µL of mass spec grade rLysC (Promega) was added to each sample and proteins were digested for 3 hours at 37°C and 700 rpm. Following the rLysC digestion, 660 µL of trypsin buffer (2 mM $CaCl_2$, 5 mM DTT, 50 mM $NH_4HCO_3$, 10% acetonitrile (ACN)) was added to each sample. After the addition of trypsin buffer, 1 µL of trypsin beads from a Poroszyme Immobilized Trypsin Cartridge (Applied Biosystems) were added to each sample. Samples were incubated with trypsin beads for 16 hours at 30°C on a rotator. After digestion, samples were stored at 4°C (briefly, not all can be desalted at once) until they could be desalted. Following digestion, peptides were desalted using OMIX C18 pipette tips (Agilent Technologies). For this, samples were centrifuged briefly to pellet the trypsin beads before desalting. Tips were activated by washing with 100 µL of methanol and then washed twice with 100 µL of 0.1 % formic acid (FA). Peptide solution was then acidified with the addition of FA to a final concentration of 3 %. The solution containing peptides was pipetted through the C18 tips and then washed twice with 100 µL of 0.1 % FA. Peptides were then eluted and saved from the C18 tips by being washed twice with 100 µL of methanol. After desalting, peptides were dried in a ScanSpeed 40 speed vacuum with a ScanVac vacuum control and stored at -20 °C.

*LC-MS/MS*

To prepare the peptides for mass spectrometry analysis, peptides were resuspended in 250 µL of 2 % acetonitrile (ACN) and 0.1 % formic acid (FA). 5 µL were then injected into an EASY-Spray C18 column (2µm, 100Å 75 µm x 50 µm, Thermo). Peptides were eluted from

the column for 150 min using a 90 min linear gradient starting from 96 % solvent A (0.1 % FA) and 4 % solvent B (80 % ACN, 0.1 % FA) to 35 % of solvent B with a flow rate of 0.3 µL/min. The linear gradient was followed by an increase of solvent B to 90 % over 1 min and held at 90 % for 8 min. Solvent B was then adjusted from 90 % to 4 % over 1 min and held at 4 % for the remaining 50 min. Ion source was an EASY-Spray source with a spray voltage of 1.9 kV. Mass spectrometry measurements were taken using an LC-QExactive-Plus (Thermo) with the following settings: 0 to 150 min; MS1: positive polarity, full scan range 380-1800 *m/z*, resolution 70,000, collision-induced dissociation (CID) fragmentation for the 20 most intense ions; MS2: loop count 20, resolution 17,500, scan range 200-2000 *m/z.*

## Data Analysis

Relevant scripts for data analysis can be found at the Github repository https://github.com/hodgskiss/Carbon_Limitation_Nviennensis.

*DNA Production, Protein Production, and Carbon Consumption*

Values for DNA (µg), protein (µg), and consumed carbon (mmol) were normalized to the total amount of nitrite produced in each culture (mmol). Normalized values were subjected to a box plot analysis as described below for protein and metabolite values. For carbon consumption some samples were removed: A1-A3 did not have GC measurements available; E2, G1, and G2 were removed as carbon consumption values were either negative (E2) or too low to be considered biologically accurate (G1 and G2), likely due to sampling errors when collecting the gas phase.

*Metabolites*

Extracted metabolites were randomly distributed between three batches to be run on the GC-MS. Quality control samples containing known concentrations of metabolites were run for each batch individually. Detected trimethylsilyl (TMS) groups were combined for each metabolite. The internal standard PGP was used to normalize sample as it showed less variation than the PE internal standard. Based on an initial analysis of metabolite areas, some contamination could be seen from blank samples that contained only purified water. Therefore, PGP normalized areas from blank samples were subtracted from samples within their perspective runs. PGP normalized and blank corrected sample values were then used to determine absolute quantification of metabolites.

Standard curves were manually created for each metabolite in each batch. In cases where concentrations on the standard curve became non-linear, only quality curve points that captured the spread of areas from samples were used (i.e. if only low concentrations were detected in samples, high values of standards were disregarded). Linear regression was applied to selected quality control points and slope and intercept values were used to obtain pmol values for metabolites.

Absolute amounts of metabolites for each culture were converted into carbon-moles (C-Moles) and used in conjunction with the amount of carbon consumed for each culture to normalize values to show how much consumed carbon was allocated for each metabolite (i.e. C-mol metabolite per C-mol carbon consumed). Carbon normalized values were subjected to a boxplot analysis for each metabolite in each condition. Based on this analysis outliers were removed. Normalized values with outliers removed were then hierarchically clustered using the heatmap.2 (25) function in R version 4.3.1 (26).

*Proteins*

Protein identification from raw mass spectrometry data was analyzed using MaxQuant version 1.6.6.0 (27). Max missed cleavages was set to 2 with variable modifications of "Oxidation (M)" and "Acetyl (Protein N-term)", fixed modifications set to "Carbamidomethyl (C)", an FDR of 0.01, and the digestive enzyme as Trypsin/P and LysC. Minimum number of unique and razor peptides was set to 2 and protein values were LFQ normalized. Reference proteome for *N. viennensis* was downloaded from Uniprot in spring of 2019.

LFQ normalized data was analyzed using a boxplot analysis for each protein in each condition. Identified outliers were removed and averages were calculated for each protein in each group. A PCA plot of the cleaned up data was made using the fviz_pca_ind function of the factoextra package (28) in R. Cleaned up and averaged data were hierarchically clustered using the heatmap.2 (25) function in R and split into 7 clusters. For mean comparisons, individual proteins were tested for normality (Shapiro test, shapiro_test() function of rstatix package (29)) and homogeneity of variance (Levene test, leveneTest() function of car package (30)). If all conditions passed these tests, one-way ANOVA (aov() function, stats package (26)) was used to determine if protein averages varied among conditions. If all conditions did not pass these tests, a Kruskal-Wallis test (Kruskal.test() function, stats package (26)) was used to determine if protein averages varied among conditions. Proteins showing a statistical difference were identified using an adjusted $P$ value $\leq 0.05$ among the tested conditions. For proteins of

interest, Tukey tests (TukeyHSD() function, stats package (26)) or Dunn's test (dunnTest() function, FSA package (31)) was used to determine which conditions were different for proteins with ANOVA and Kruskal-Wallis analysis respectively. In each case, *P* values were adjusted using the Benjamini-Hochberg method (p.adjust() function, method="BH", stats package (26)).

Clusters of interest for the most limited condition (E; Figure 3, Cluster V ) and non-limited conditions (ACDG; Figure 4, Cluster VII) were identified by choosing the clusters with the highest average values for condition E and ACDG respectively.

Proteins were evaluated based on functional categories according to their archaeal clusters of orthologous groups (arCOG) categories as defined in Reyes et al. (2020) (32). For arCOG enrichment analysis, the phyper() function of the stats package (26) was used for defined clusters or groups of proteins and compared against all detected proteins in the dataset with the parameter lower.tail=FALSE. *P* values for each group of proteins were adjusted using the Benjamini-Hochberg method using the p.adjust() function of the stats R package (26).

*Statistical Data Analysis*

Clusters of high relative abundance for metabolites in condition E (Figure 5, Cluster Met-IV) were correlated with all proteins across all conditions to identify trends in metabolism. The R function cor.test was used with method set to "pearson" and use set to "complete.obs". *P* values were adjusted according to the Benjamini-Hochberg method.

A correlation analysis was performed with all proteins for all conditions against MsrA to identify other proteins with similar patterns. The R function cor.test was used with method set to "pearson" and use set to "complete.obs". *P* values were adjusted according to the Benjamini-Hochberg method.

Correlation analyses for specific metabolites (glucose, trehalose, maltose, and melibiose) were performed across all proteins and conditions. The R function cor.test was used with method set to "pearson" and use set to "complete.obs". *P* values were adjusted according to the Benjamini-Hochberg method.

Other R packages used in analysis included ggplot2 (33), dplyr (34), reshape2 (35), tidyverse (36), ggVennDiagram (37), RColorBrewer (38), expss (39), magrittr (40), plot.matrix (41), and viridis (42).

*Partial Least Squres-Discriminant Analysis (PLS-DA)*

A PLS-DA analysis was performed with all proteins to identify specific proteins of interest under carbon limitation. Outlier protein values were replaced with averages of the respective protein for the respective condition. Protein data was then scaled using the pareto method (done for each protein across all conditions). The mixOmics R package (43) was used with the plsda command (X as the scaled proteins, Y a vector representing each condition, scale=FALSE) to create a PLS-DA plot. The function vip() was used to identify VIP (variable importance in projection) scores for proteins pertaining to component 1 which separated samples by carbon limitation. The protein with the highest VIP score in component 1 was A0A060HNZ6 (NVIE_010650), a hypothetical protein.

*Identification of NVIE_010650*

A blast search of NVIE_010650 revealed no hits with putative functions. A structural search using the AlphaFold generated structure was performed using Foldseek (44). The hits with putative function were summarized (Dataset_S1). The structures of the retrieved sequences together with that of NVIE_010650 were aligned using PROMALS3D (45). The alignment was then filtered with trimAl (46) and trees were constructed with IQ-TREE v2.3.6 (47) and visualized in iTOL v.6 (48).

# Supplementary Figures

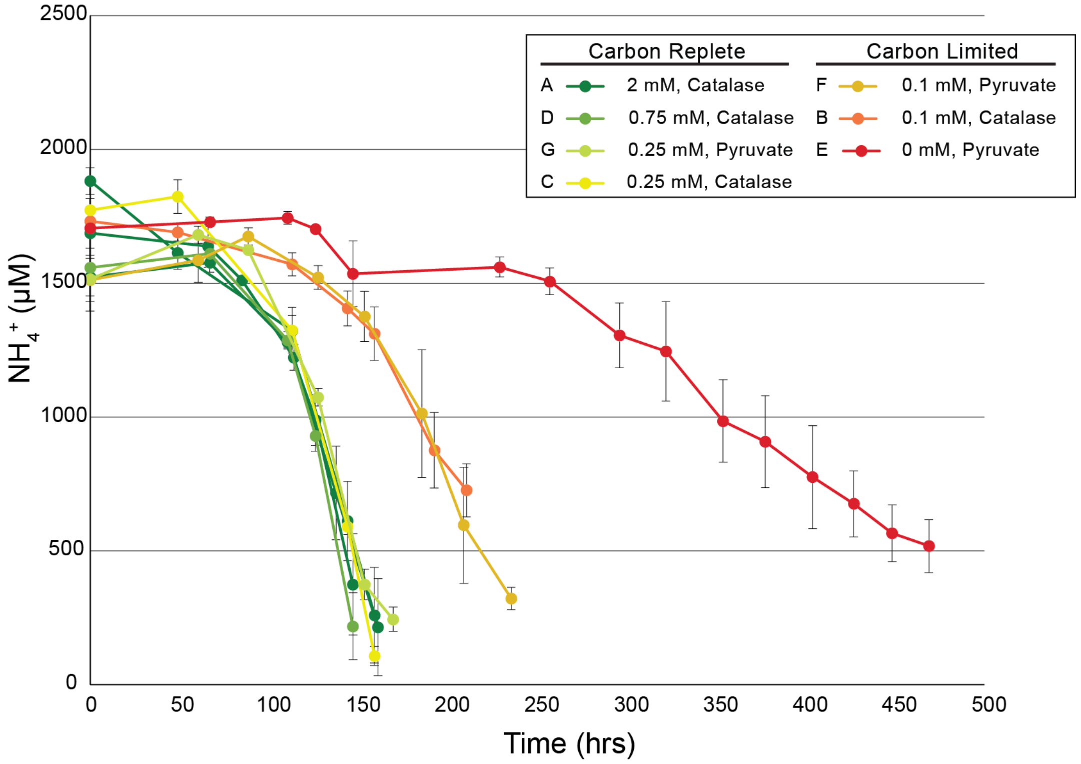


**Figure S1: Consumption of ammonium during growth.** Error bars represent standard deviations of biological replicates.

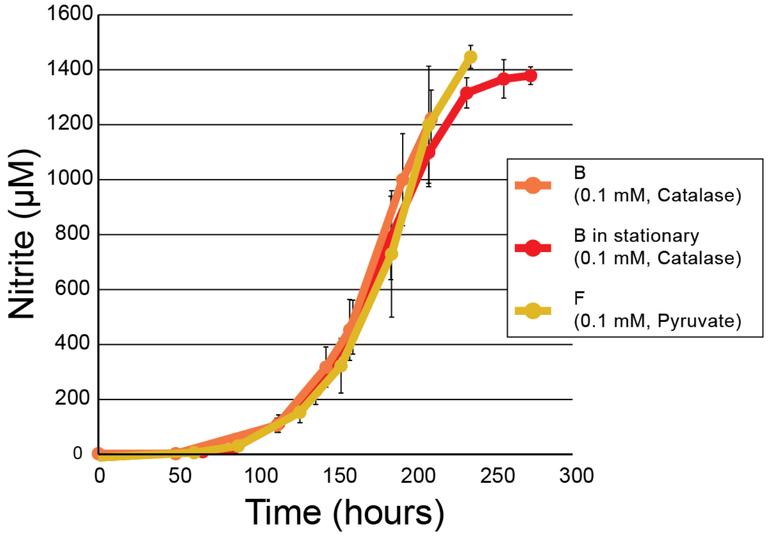


**Figure S2: Growth curves of conditions B and F.** Growth curves showing condition F (0.1 mM carbon with pyruvate) and condition B (0.1 mM carbon with catalase). "B in stationary" represents condition B when allowed to grow above ~1200-1300 mM nitrite. At this point, the cultures begin to enter stationary phase. Condition F is able to oxidize more ammonia without entering stationary phase.

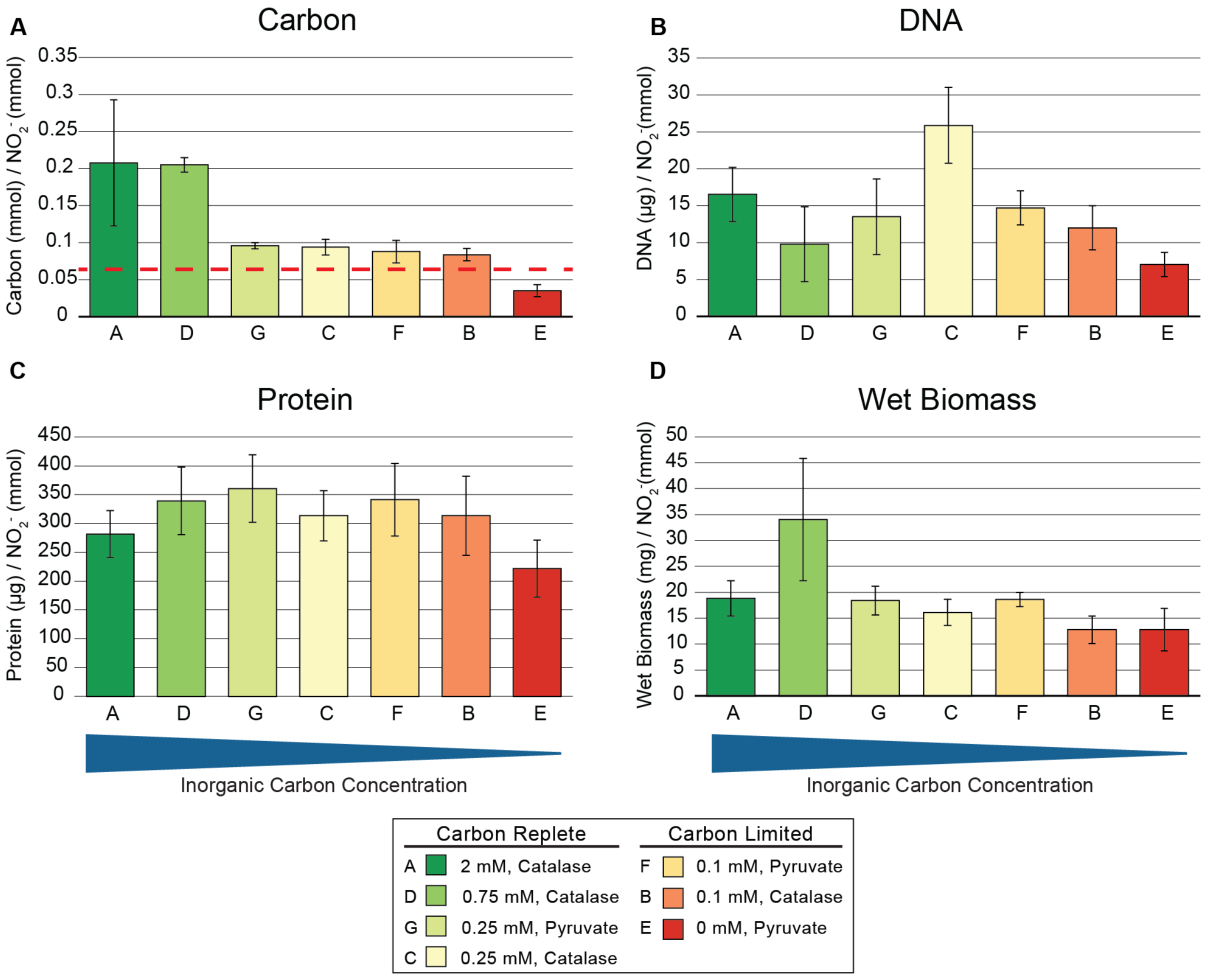


**Figure S3: Carbon and biomass data of *Nitrososphaera viennensis*. A.)** Average inorganic carbon consumption (see Carbon Balance in Supp. Materials and Methods) normalized to nitrite production. The red dotted line shows the theoretical amount of carbon consumption for 1 mmol of ammonia consumed based on Equation 18. **B.)** Average DNA content normalized to nitrite produced. **C.)** Average protein content normalized to nitrite production. **D.)** Average wet biomass produced normalized to nitrite production. Error bars represent standard deviations of cultures.

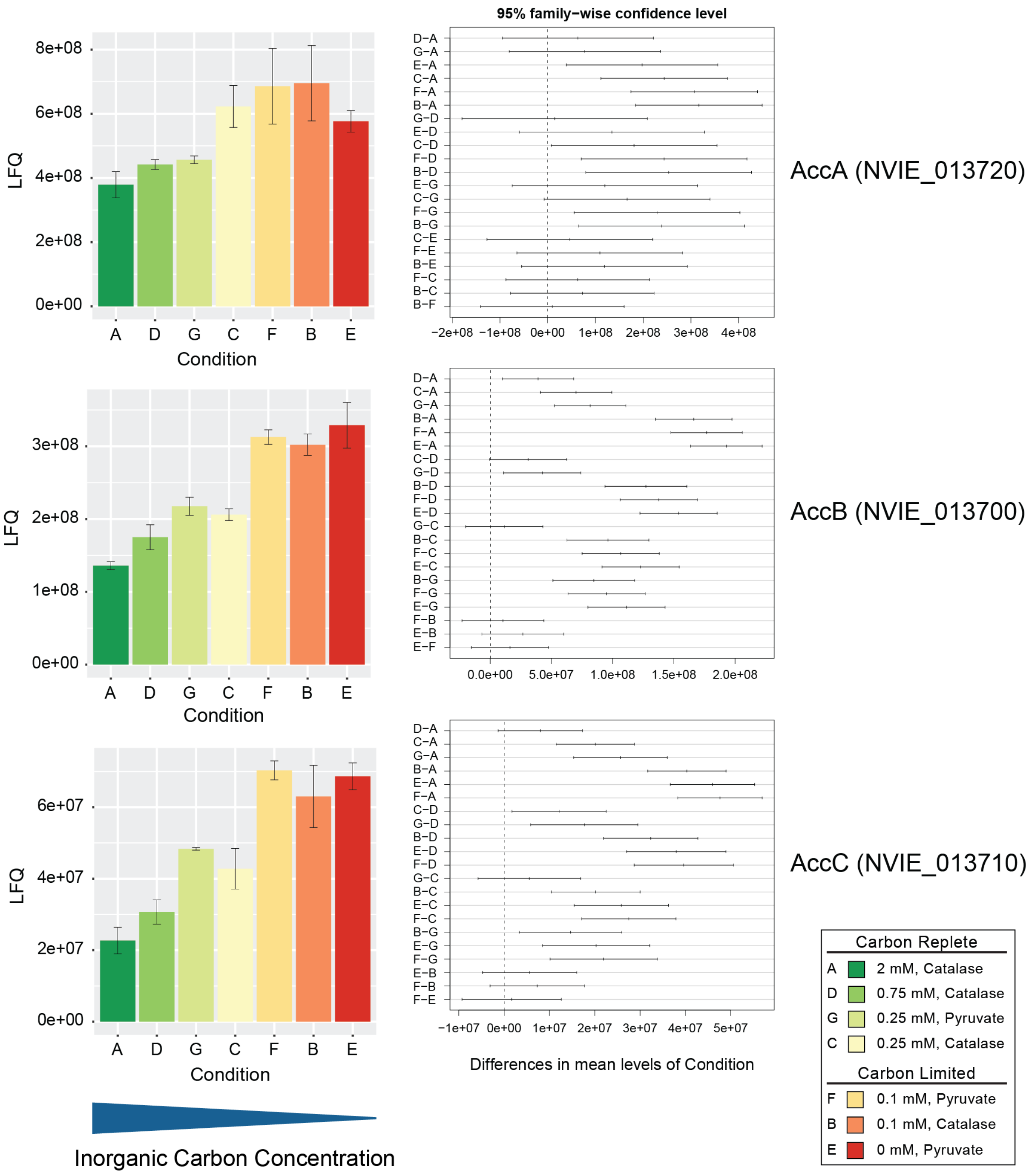


**Figure S4: Relative intensities and 95% confidence intervals of subunits of acetyl-CoA carboxylase (Acc) across conditions.** Average intensities are shown as normalized label-free quantification (LFQ) values. Error bars represent standard deviations. Family-wise confidence intervals represent differences between compared conditions. Confidence intervals that do not cross 0 represent statistically significant differences between the two tested groups based on a post hoc Tukey Test.

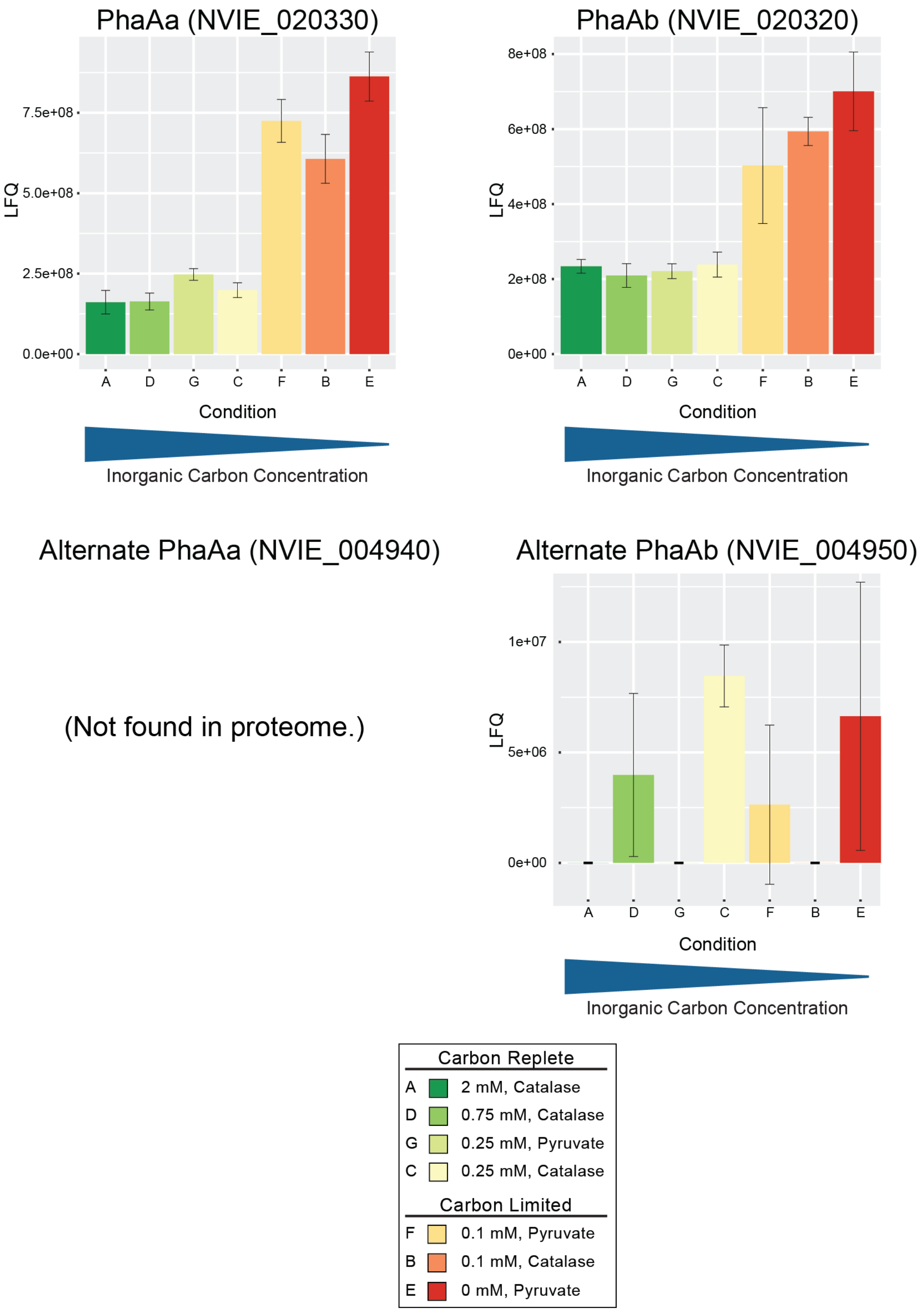


**Figure S5: Relative intensities of subunits of candidates for acetoacetyl-CoA thiolase subunits (PhaAab).** Average intensities are shown as normalized label-free quantification (LFQ) values. Error bars represent standard deviations.

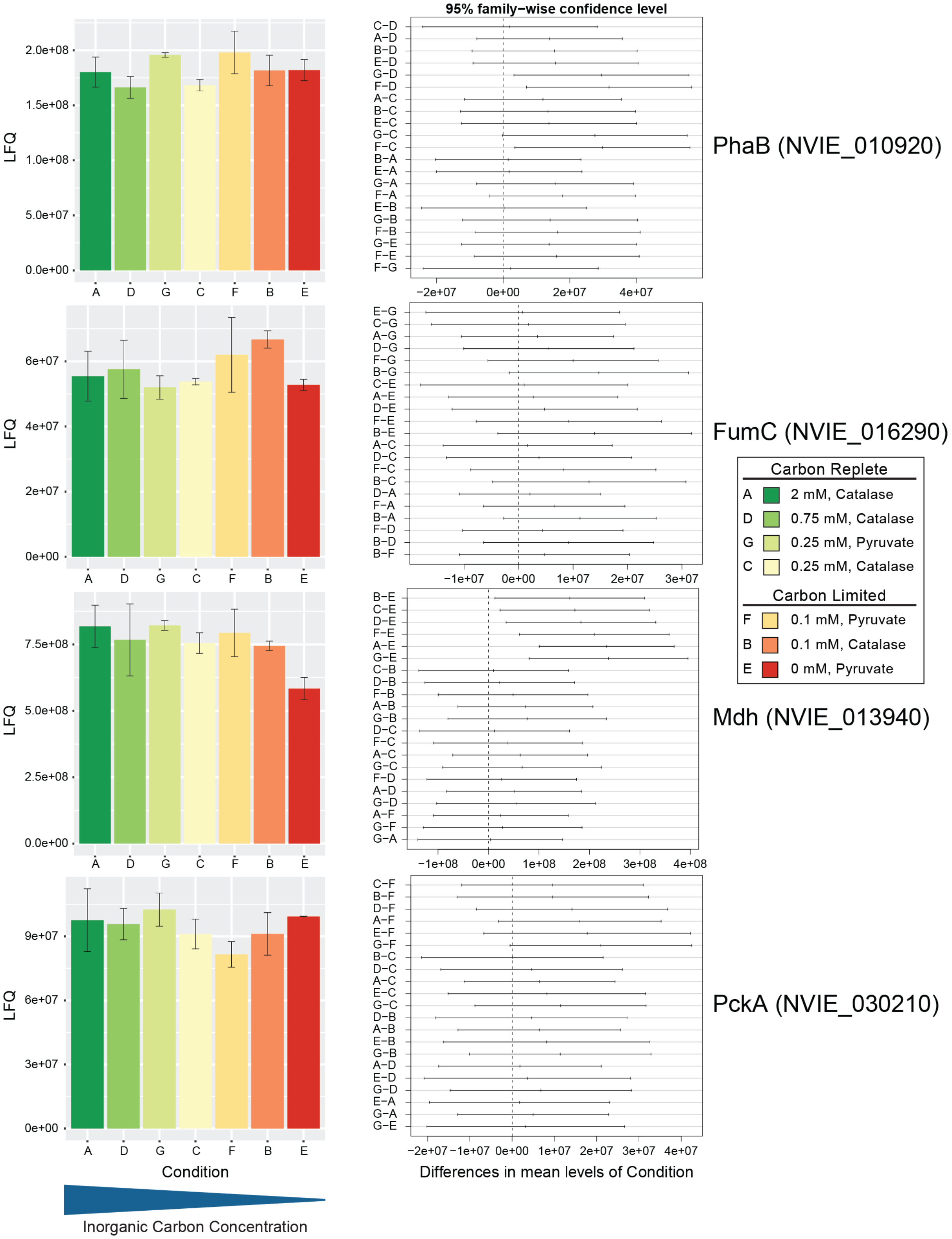


**Figure S6: Proteins of the central carbon metabolism that are not up- or down-regulated under carbon limitation.** Up-regulated and down-regulated proteins are defined as proteins found within Cluster V and Cluster VII respectively that show a statistical difference between condition E and condition A (see Figure 4). Average intensities are shown as normalized label free quantification (LFQ) values. Error bars represent standard deviations. Family-wise confidence intervals represent differences between compared conditions. Confidence intervals that do not cross 0 represent statistically significant differences between the two tested groups based on a post hoc Tukey Test.

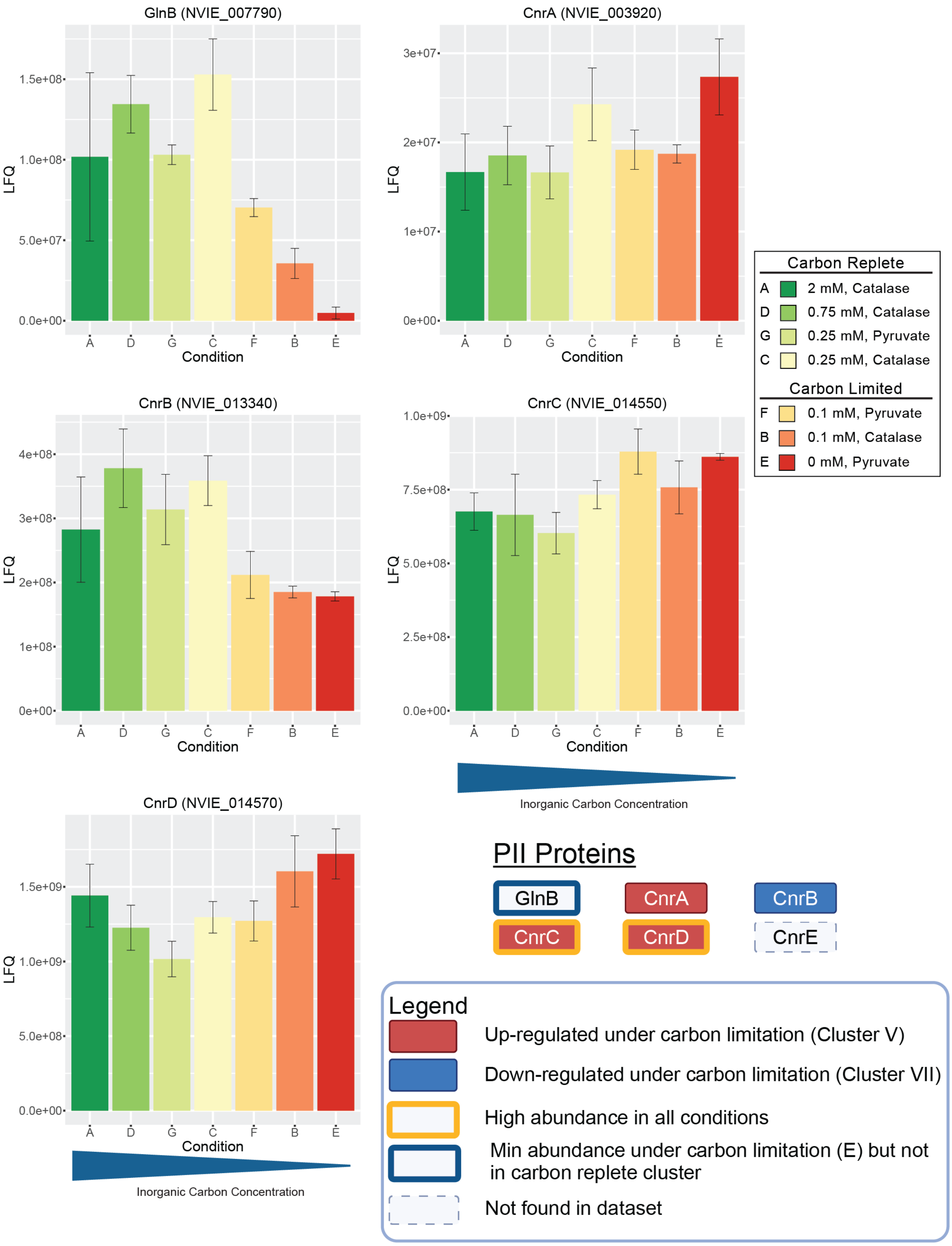


**Figure S7: PII protein response to carbon limitation.** Average intensities are shown as normalized label-free quantification (LFQ) values. Error bars represent standard deviations. Some pieces created in BioRender. Hodgskiss, L. (2025) https://BioRender.com/j28f691.

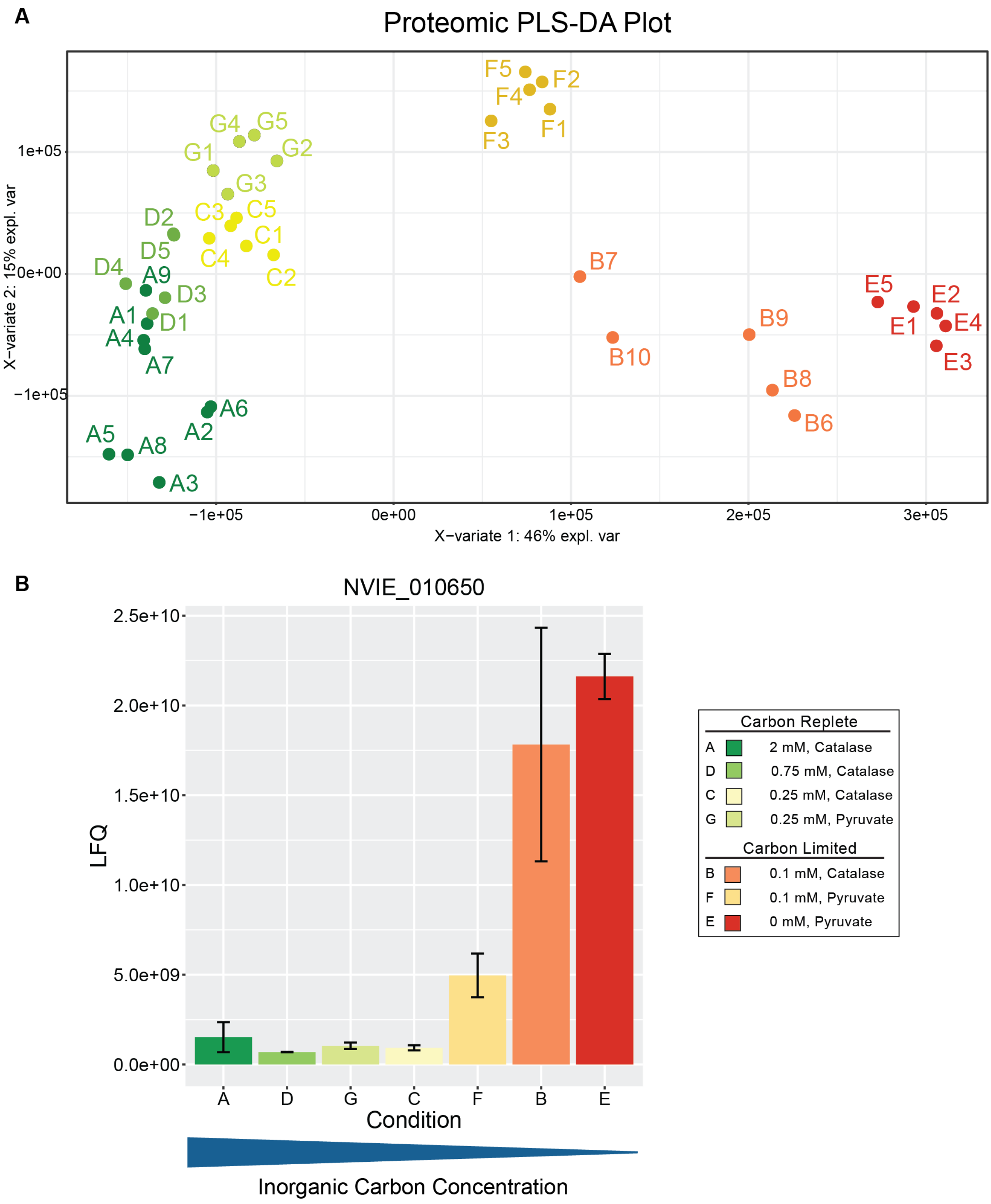


**Figure S8: PLS-DA analysis. A.)** Plot of a supervised partial least squares discriminant analysis (PLS-DA). **B.)** Average intensities of the protein with the strongest VIP value corresponding with X-variate 1 of the PLS-DA analysis. Average intensities are shown as normalized label-free quantification (LFQ) values. Error bars represent standard deviations.

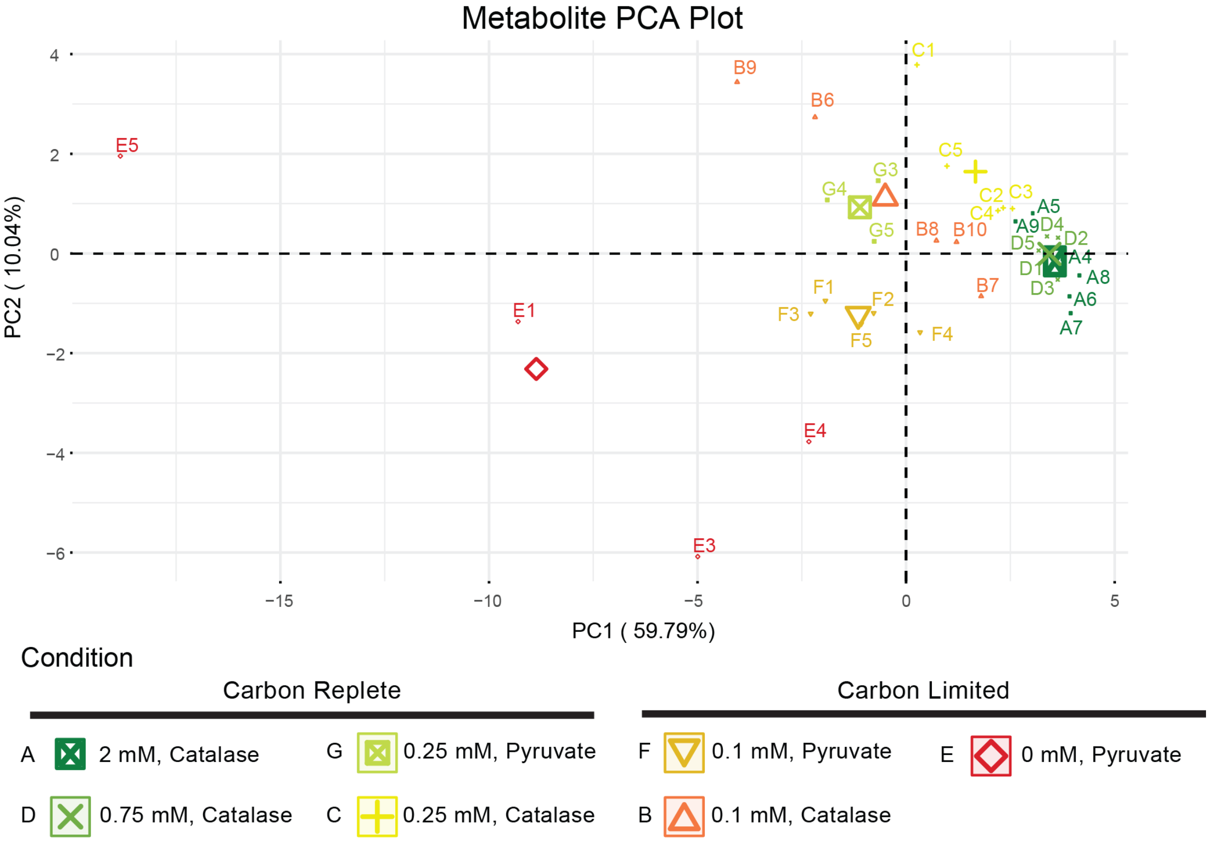


**Figure S9: Principal component analysis of *Nitrososphaera viennensis* metabolomes.** Bold and enlarged points represent centers of points within respective conditions.

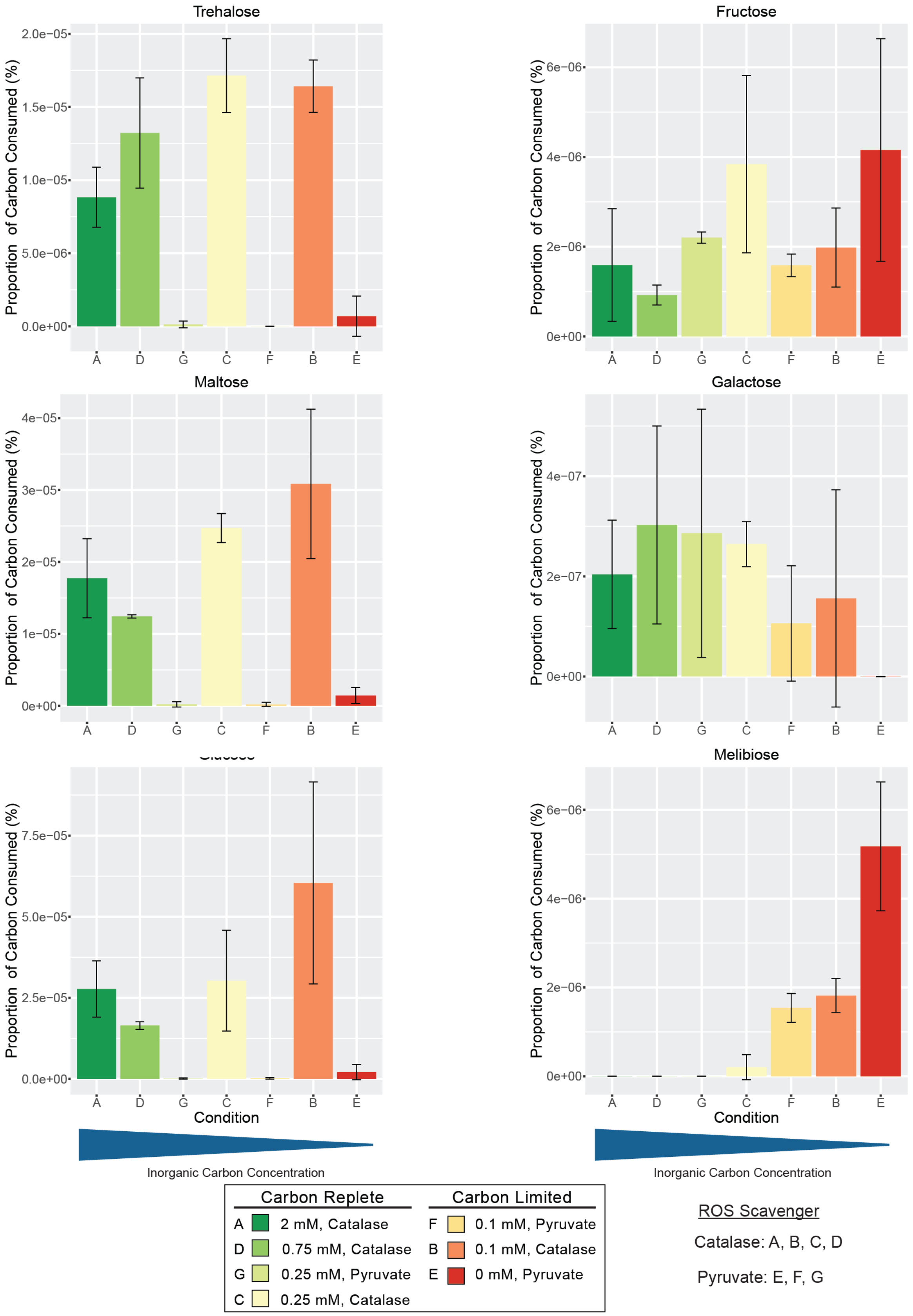


**Figure S10: Sugar abundance across carbon conditions.** Average abundance values of quantified sugars normalized to amount of carbon consumed in each culture. Error bars represent standard deviations.

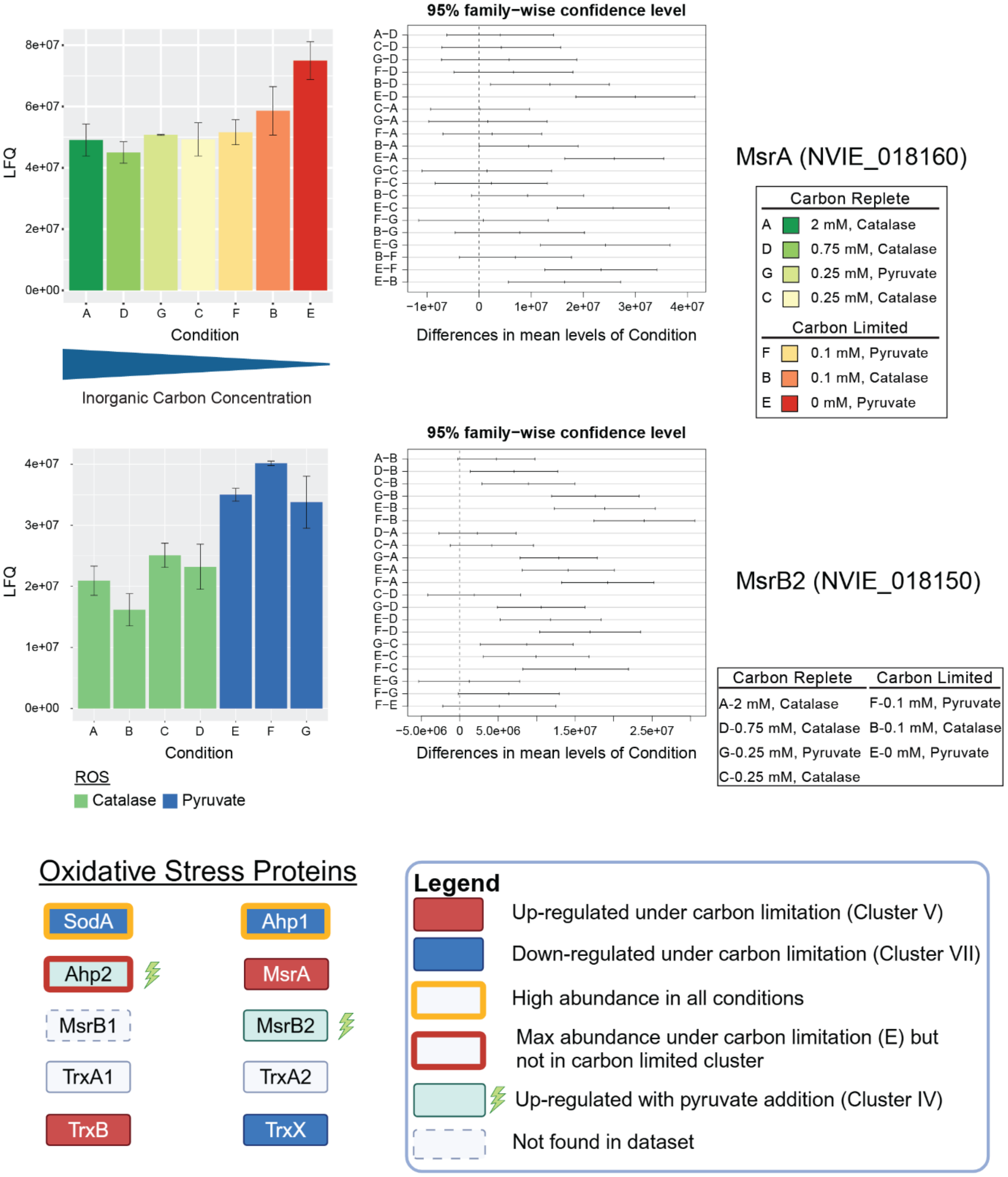


**Figure S11: Oxygen detoxification proteins in *N. viennensis*.** Average intensities are shown as normalized label-free quantification (LFQ) values. Error bars represent standard deviations. Family-wise confidence intervals represent differences between compared conditions. Confidence intervals that do not cross 0 represent statistically significant differences between the two tested groups based on a post hoc Tukey Test. Locus tags and accession numbers can be found in Dataset S1. Some pieces created in BioRender. Hodgskiss, L. (2025) https://BioRender.com/u08b790.

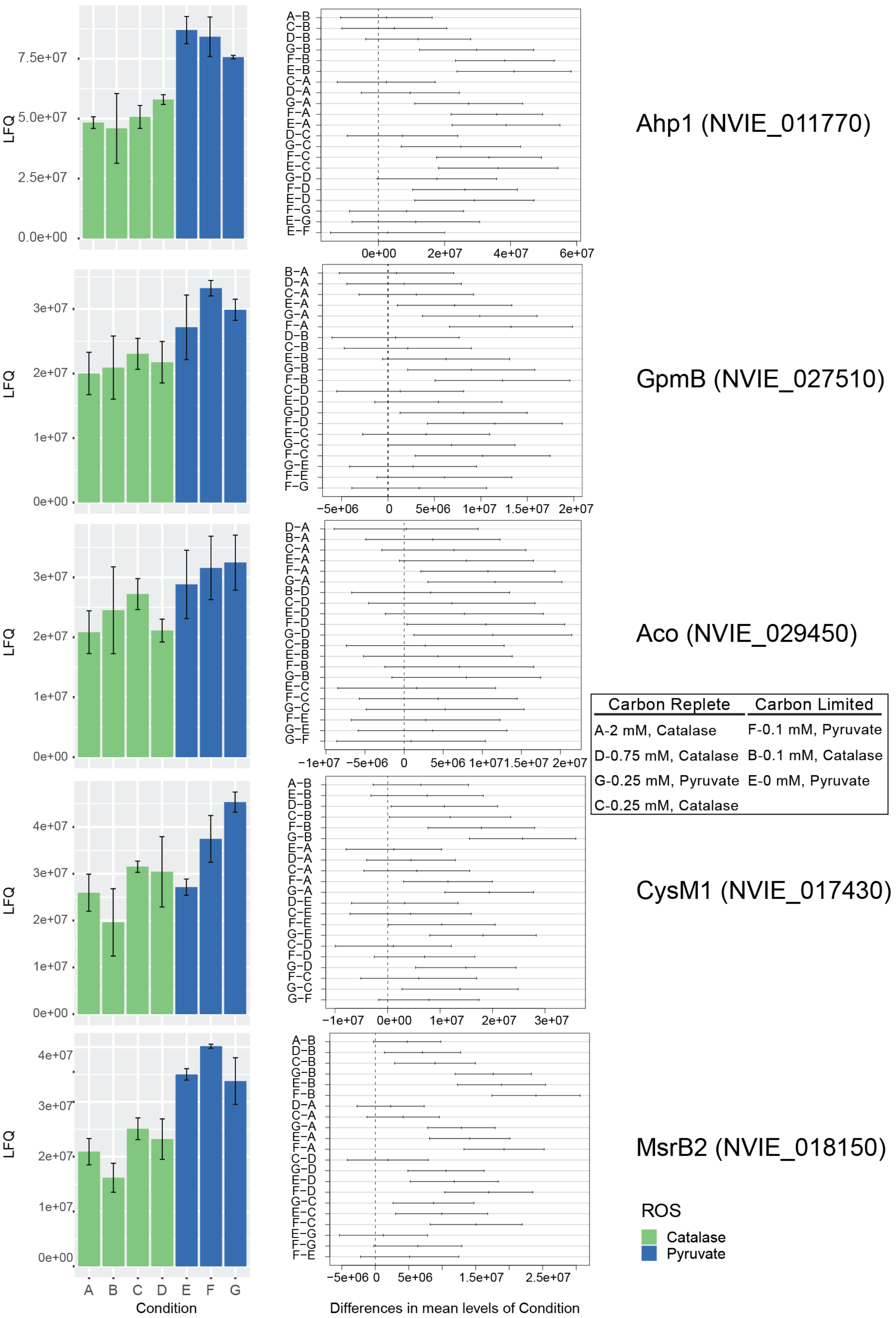


**Figure S12: Central carbon and oxygen detoxification proteins that react to different ROS scavengers.** Average intensities are shown as normalized label-free quantification (LFQ) values. Error bars represent standard deviations. Family-wise confidence intervals represent differences between compared conditions. Confidence intervals that do not cross 0 represent statistically significant differences between the two tested groups based on a post hoc Tukey Test.

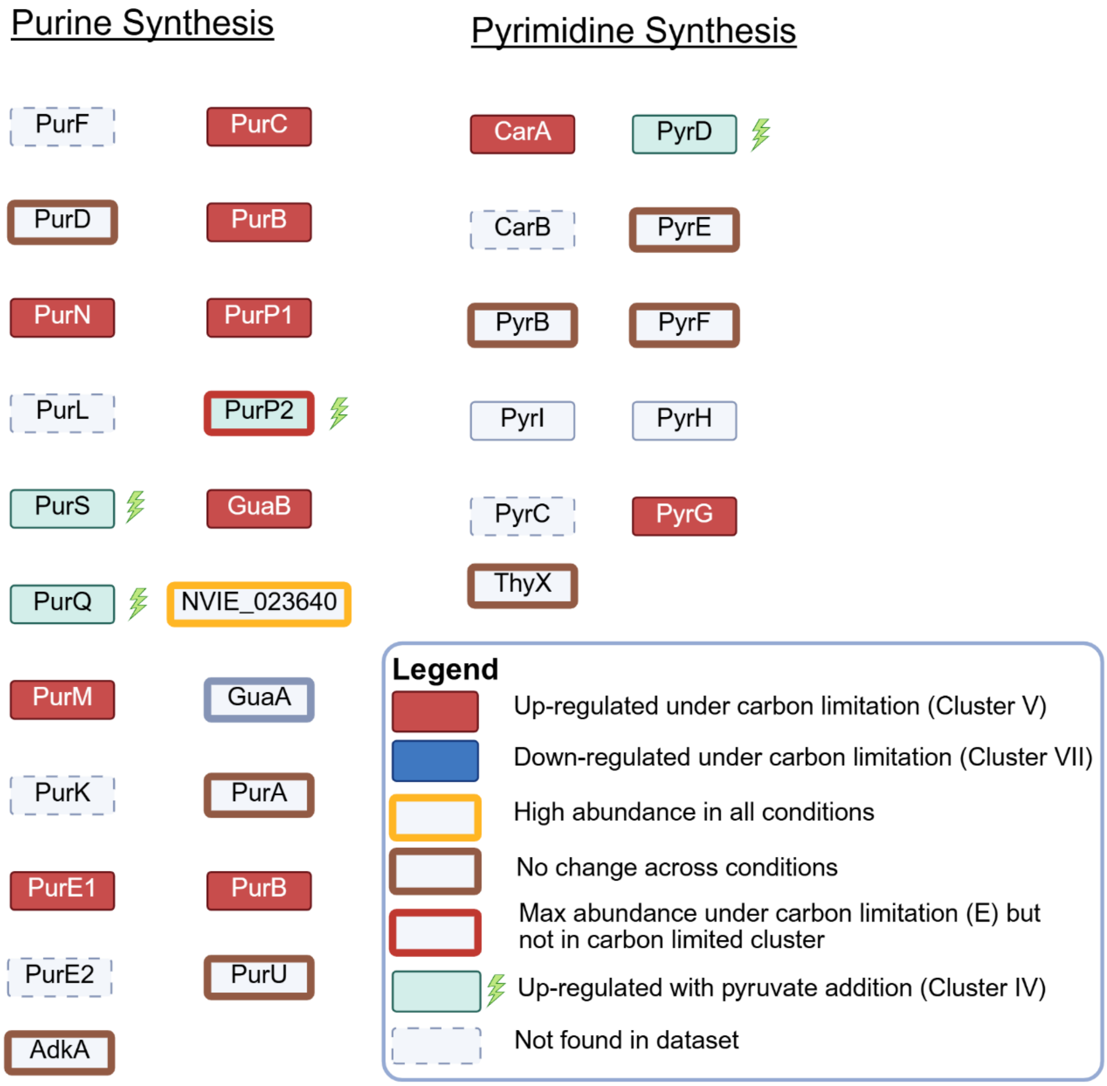


**Figure S13: Purine and pyrimidine synthesis proteins in *N. viennensis*.** Locus tags and accession numbers can be found in Dataset S1. Created in BioRender. Hodgskiss, L. (2025) https://BioRender.com/c49z928.

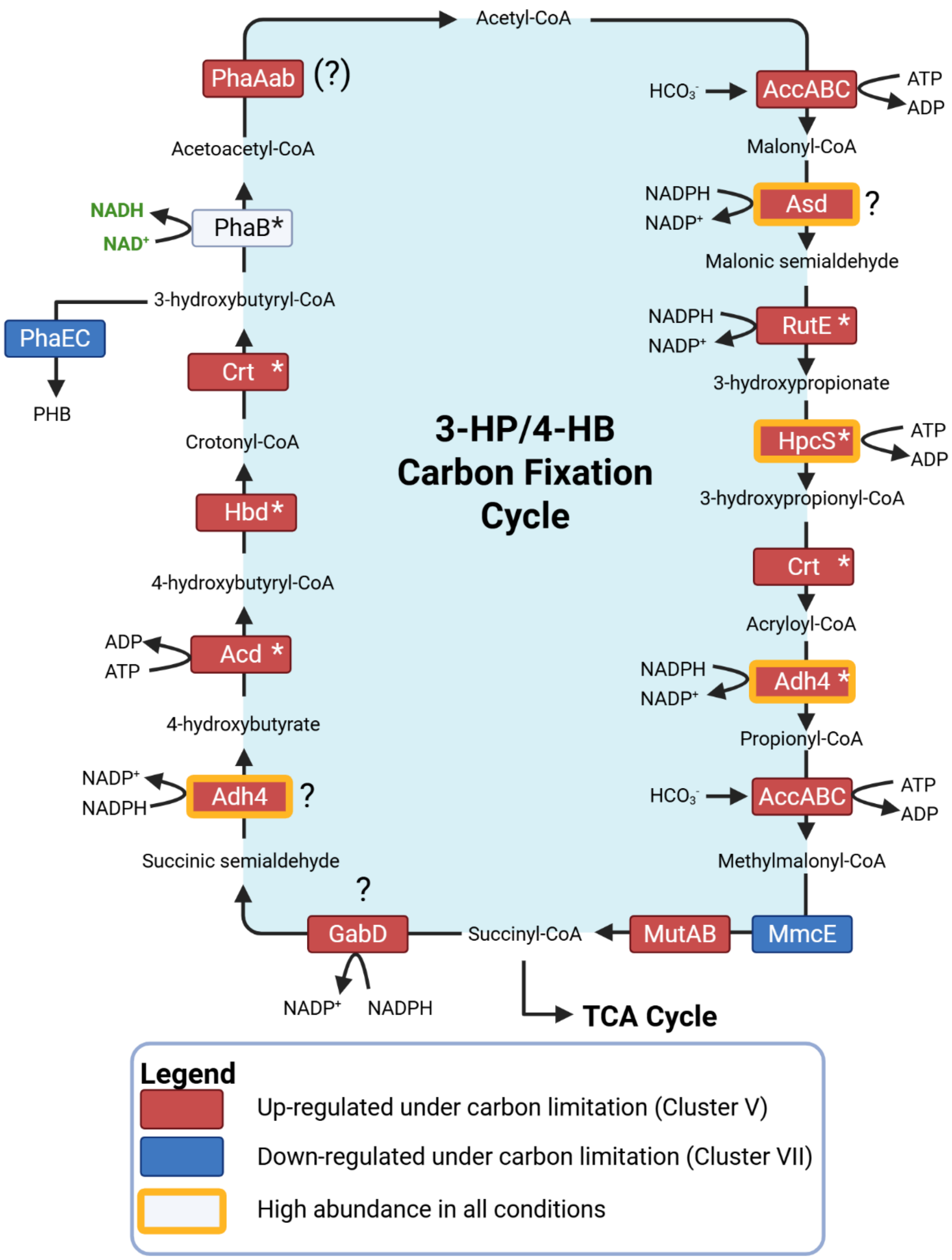


**Figure S14: Detailed 3-hydroxypropionate/4-hydroxybutyrate (3-HP/4-HB) cycle in *Nitrososphaera viennensis*.** Up-regulated and down-regulated proteins are defined as proteins found within Cluster V and Cluster VII respectively that show a statistical difference between condition E and condition A (see Figure 4). Asterisks denote characterized proteins from AOA of the carbon fixation cycle (see Dataset_S1). Question marks denote steps lacking strong evidence for protein candidates (Dataset S1). Question mark with brackets indicate proteins with strong bioinformatic evidence but with multiple candidates. Locus tags and accession numbers for proteins can be found in Dataset S1. Created in BioRender. Hodgskiss, L. (2025) https://BioRender.com/1tde9v3.

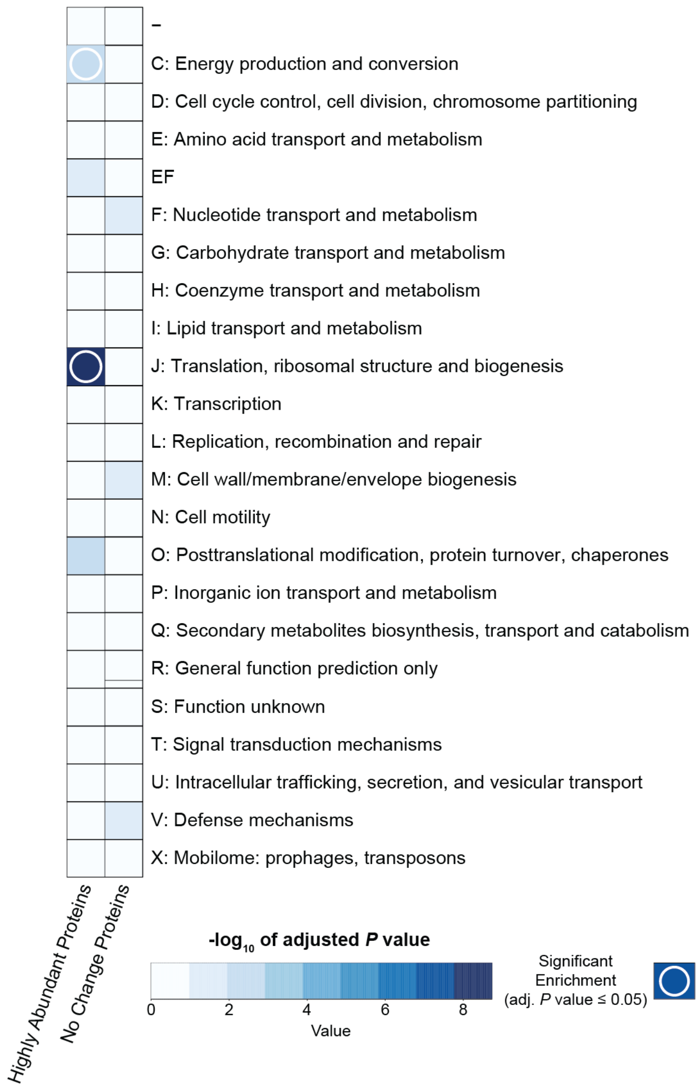


**Figure S15: Enrichment analysis of archaeal clusters of orthologous genes (arCOG) categories in highly abundant proteins and proteins that do not change between different conditions.** Boxes with a white circle indicate arCOG categories that are enriched in their respective cluster based off of a hypergeometric test.

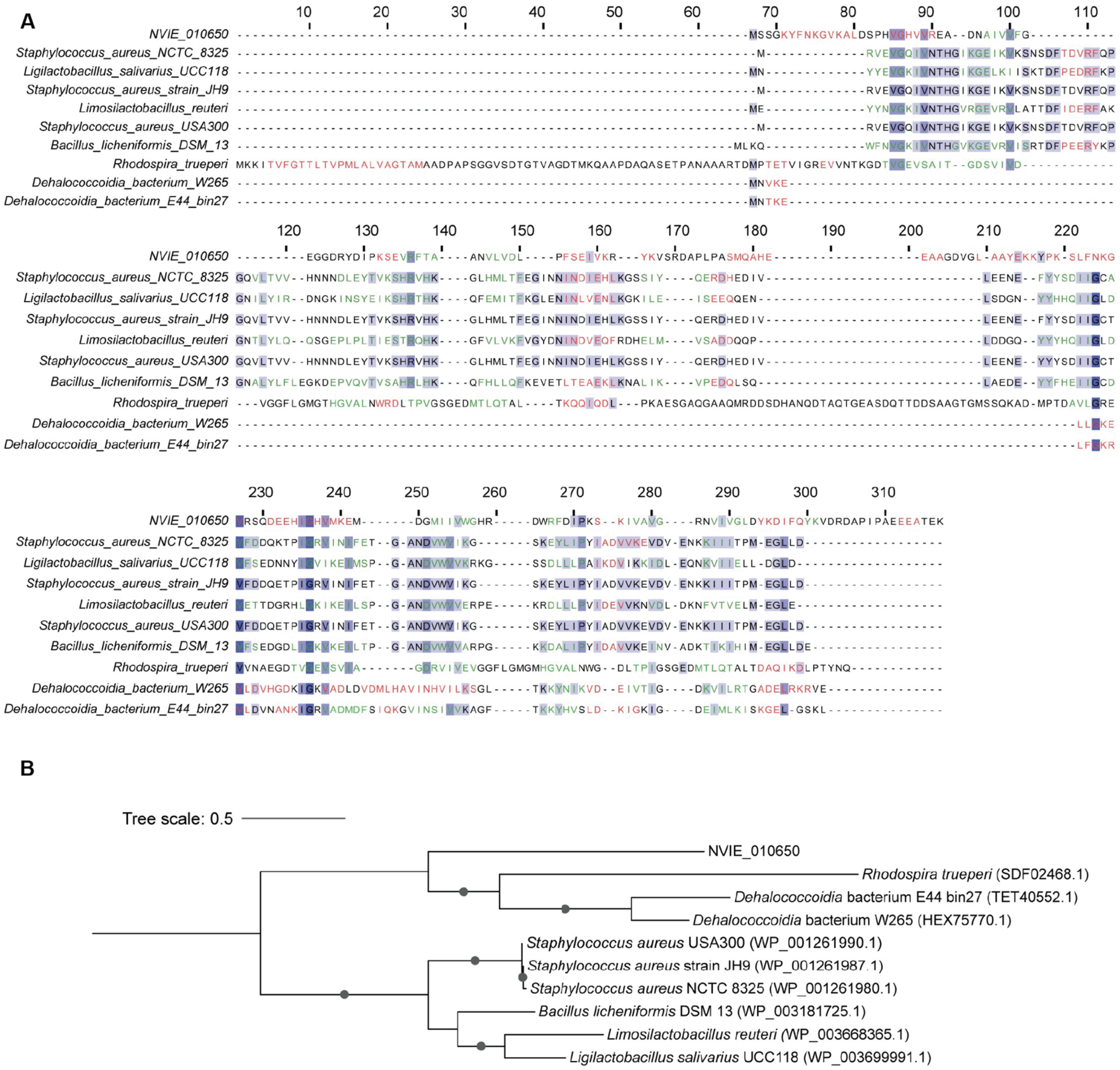


**Figure S16: Analysis of structural search matches for NVIE_010650. A.)** Amino acid alignments of NVIE_010650 with closest structural search matches from FoldSeek. **B.)** Amino acid tree showing the relatedness of NVIE_010650 with closest structural matches from FoldSeek.

**References**


1. Makarova KS, Wolf YI, Koonin EV. 2015. Archaeal Clusters of Orthologous Genes (arCOGs): an update and application for analysis of shared features between Thermococcales, Methanococcales, and Methanobacteriales. Life Basel Switz 5:818–40.

2. Hodgskiss LH, Melcher M, Kerou M, Chen W, Ponce-Toledo RI, Savvides SN, Wienkoop S, Hartl M, Schleper C. 2023. Unexpected complexity of the ammonia monooxygenase in archaea. ISME J 17:588–599.

3. Kerou M, Offre P, Valledor L, Abby SS, Melcher M, Nagler M, Weckwerth W, Schleper C. 2016. Proteomics and comparative genomics of Nitrososphaera viennensis reveal the core genome and adaptations of archaeal ammonia oxidizers. Proc Natl Acad Sci U S A 113:E7937–E7946.

4. Gerhardt ECM, Rodrigues TE, Müller-santos M, Pedrosa FO, Souza EM, Forchhammer K, Huergo LF. 2015. The bacterial signal transduction protein GlnB regulates the committed step in fatty acid biosynthesis by acting as a dissociable regulatory subunit of acetyl-CoA carboxylase. Mol Microbiol 95:1025–1035.

5. Feria Bourrellier AB, Valot B, Guillot A, Ambard-Bretteville F, Vidal J, Hodges M. 2010. Chloroplast acetyl-CoA carboxylase activity is 2-oxoglutarate–regulated by interaction of PII with the biotin carboxyl carrier subunit. Proc Natl Acad Sci U S A 107:502–507.

6. Hauf W, Schmid K, Gerhardt ECM, Huergo LF, Forchhammer K. 2016. Interaction of the nitrogen regulatory protein GlnB (PII) with biotin carboxyl carrier protein (BCCP) controls acetyl-CoA levels in the cyanobacterium *Synechocystis* sp. PCC 6803. Front Microbiol 7.

7. Nußbaum P, Kureisaite-Ciziene D, Bellini D, van der Does C, Kojic M, Taib N, Yeates A, Tourte M, Gribaldo S, Loose M, Löwe J, Albers S-V. 2024. Proteins containing photosynthetic reaction centre domains modulate FtsZ-based archaeal cell division. Nat Microbiol 9:698–711.

8. Anantharaman V, Aravind L. 2002. The PRC-barrel: a widespread, conserved domain shared by photosynthetic reaction center subunits and proteins of RNA metabolism. Genome Biol 3:research0061.1.

9. Pelve EA, Lindås AC, Martens-Habbena W, de la Torre JR, Stahl DA, Bernander R. 2011. Cdv-based cell division and cell cycle organization in the thaumarchaeon *Nitrosopumilus maritimus*. Mol Microbiol 82:555–566.

10. Tikhomirova A, Rahman MM, Kidd SP, Ferrero RL, Roujeinikova A. 2024. Cysteine and resistance to oxidative stress: implications for virulence and antibiotic resistance. Trends Microbiol 32:93–104.

11. Abby SS, Kerou M, Schleper C. 2020. Ancestral reconstructions decipher major adaptations of ammonia-oxidizing archaea upon radiation into moderate terrestrial and marine environments. mBio 11:e02371-20.

12. Castro L, Rodriguez M, Radi R. 1994. Aconitase is readily inactivated by peroxynitrite, but not by its precursor, nitric oxide. J Biol Chem 269:29409–29415.

13. Tourna M, Stieglmeier M, Spang A, Könneke M, Schintlmeister A, Urich T. 2011. Nitrososphaera viennensis, an ammonia oxidizing archaeon from soil. Proc Natl Acad Sci USA 108:8420–8425.

14. Kim J-G, Park S-J, Sinninghe Damsté JS, Schouten S, Rijpstra WIC, Jung M-Y, Kim S-J, Gwak J-H, Hong H, Si O-J, Lee S, Madsen EL, Rhee S-K. 2016. Hydrogen peroxide detoxification is a key mechanism for growth of ammonia-oxidizing archaea. Proc Natl Acad Sci U S A 113:7888–93.

15. Bei Q, Reitz T, Schädler M, Hodgskiss LH, Peng J, Schnabel B, Buscot F, Eisenhauer N, Schleper C, Heintz-Buschart A. 2024. Metabolic potential of *Nitrososphaera*-associated clades. ISME J 18:wrae086.

16. McCarty PL. 2006. Thermodynamic electron equivalents model for bacterial yield prediction: modifications and comparative evaluations. Biotechnol Bioeng 97:377–388.

17. Suzuki I, Dular U, Kwok SC. 1974. Ammonia or ammonium ion as substrate for oxidation by *Nitrosomonas europaea* cells and extracts. J Bacteriol 120:556–558.

18. Rittmann BE, McCarty PL. 2001. Environmental Biotechnology: Principles and ApplicationsIndian Edition. McGraw Hill Education (India) Private Limited.

19. Tinoco IJr, Sauer K, Wang JC, Puglisi JD, Harbison G, Rovnyak D. 2014. Physcial Chemistry Principles and Applications in Biological Sciences. Pearson, Boston.

20. Wagman DD, Evans WH, Parker VB, Halow I, Baily SM, Schumm RH. 1968. Selected Values of Chemical Thermodynamic Properties Table for the First Thirty-Four Elements in the Standard Order of Arrangement; Technical Note 270-3. U.S. Department of Commerce Insitute for Basic Standards.

21. McCarty PL. 1971. Energetics and Bacterial Growth, p. . *In* Faust, SD, Hunter, JV (eds.), Organic Compounds in Aquatic Environments. Marcel Dekker, New York.

22. Boyer G. 2024. pyCHNOSZ: Python wrapper for the thermodynamic package CHNOSZ (v0.8.15) (v0.8.15).

23. Dick JM. 2019. CHNOSZ: thermodynamic calculations and diagrams for geochemistry. Front Earth Sci 7.

24. Ott E, Fuchs FM, Moeller R, Hemmersbach R, Kawaguchi Y, Weckwerth W, Milojevic T. 2019. Molecular response of *Deinococcus radiodurans* to simulated microgravity explored by proteometabolomic approach. Sci Rep 9:18462.

25. Warnes GR, Bolker B, Bonebakker L, Gentleman R, Huber W, Liaw A, Lumley T, Maechler M, Magnusson A, Moeller S, Schwartz M, Venables B. 2020. gplots: Various R Programming Tools for Plotting Data. R package version 3.1.1.

26. R Core Team (2023). 2023. R: A language and environment for statistical computing. R Foundation for Statistical Computing, Vienna, Austria.

27. Tyanova S, Temu T, Cox J. 2016. The MaxQuant computational platform for mass spectrometry-based shotgun proteomics. Nat Protoc 11:2301–2319.

28. Kassambara A, Mundt F. 2020. factoextra: Extract and Visualize the Results of Multivariate Data Analyses.

29. Kassambara A. 2021. rstatix: Pipe-Friendly Framework for Basic Statistical Tests.

30. Fox J, Weisberg S. 2019. An R Companion to applied regression, Third Edition. Thousand Oaks CA: Sage.

31. Ogle DH, Wheeler P, Dinno A. 2021. FSA: Fisheries Stock Analysis.

32. Reyes C, Hodgskiss LH, Kerou M, Pribasnig T, Abby SS, Bayer B, Kraemer SM, Schleper C. 2020. Genome wide transcriptomic analysis of the soil ammonia oxidizing archaeon Nitrososphaera viennensis upon exposure to copper limitation. ISME J 14:2659–2674.

33. Wickham H. 2016. ggplot2: Elegant Graphics for Data Analysis. Springer-Verlag New York.

34. Wickham H, François R, Henry L, Müller K, Vaughan D, Posit Software, PBC. 2023. dplyr: a grammar of data manipulation.

35. Wickham H. 2007. Reshaping Data with the reshape Package. J Stat Softw 2112 1-20.

36. Wickham H, Averick M, Bryan J, Chang W, McGowan LD, François R, Grolemund G, Hayes A, Henry L, Hester J, Kuhn M, Pedersen TL, Miller E, Bache SM, Müller K, Ooms J, Robinson D, Seidel DP, Spinu V, Takahashi K, Vaughan D, Wilke C, Woo K, Yutani H. 2019. Welcome to the tidyverse. J Open Source Softw 443 1686.

37. Gao C-H. 2021. ggVennDiagram: a "ggplot2" implement of Venn diagram.

38. Neuwirth E. 2014. RColorBrewer: ColorBrewer Palettes.

39. Demin G. 2020. expss: tables, labels and some useful functions from spreadsheets and "SPSS" statistics.

40. Bache SM, Wickham H. 2022. magrittr: a forward-pipe operator for R.

41. Klinke S. 2021. plot.matrix: visualizes a matrix as heatmap.

42. Garnier S, Ross N, Rudis R, Camargo AP, Sciaini M, Scherer C. 2021. Rvision - colorblind-friendly color maps for R.

43. Rohart F, Gautier B, Singh A, Cao K-AL. 2017. mixOmics: An R package for 'omics feature selection and multiple data integration. PLOS Comput Biol 13:e1005752.

44. van Kempen M, Kim SS, Tumescheit C, Mirdita M, Lee J, Gilchrist CLM, Söding J, Steinegger M. 2024. Fast and accurate protein structure search with Foldseek. Nat Biotechnol 42:243–246.

45. Pei J, Kim B-H, Grishin NV. 2008. PROMALS3D: a tool for multiple protein sequence and structure alignments. Nucleic Acids Res 36:2295–2300.

46. Capella-Gutiérrez S, Silla-Martínez JM, Gabaldón T. 2009. trimAl: a tool for automated alignment trimming in large-scale phylogenetic analyses. Bioinformatics 25:1972–1973.

47. Minh BQ, Schmidt HA, Chernomor O, Schrempf D, Woodhams MD, Von Haeseler A, Lanfear R. 2020. IQ-TREE 2: new models and efficient methods for phylogenetic inference in the genomic era. Mol Biol Evol 37:1530–1534.

48. Letunic I, Bork P. 2024. Interactive Tree of Life (iTOL) v6: recent updates to the phylogenetic tree display and annotation tool. Nucleic Acids Res 52:W78–W82.
